# The ancestral levels of transcription and the evolution of sexual phenotypes in filamentous fungi

**DOI:** 10.1371/journal.pgen.1006867

**Published:** 2017-07-13

**Authors:** Frances Trail, Zheng Wang, Kayla Stefanko, Caitlyn Cubba, Jeffrey P. Townsend

**Affiliations:** 1 Department of Plant Biology, Michigan State University, East Lansing, MI, United States of America; 2 Department of Plant, Soil and Microbial Sciences, Michigan State University, East Lansing, MI, United States of America; 3 Department of Biostatistics, Yale University, New Haven, CT, United States of America; 4 Department of Ecology and Evolutionary Biology, Yale University, New Haven, CT, United States of America; 5 Program in Computational Biology and Bioinformatics, Yale University, New Haven, CT, United States of America; Stanford University, UNITED STATES

## Abstract

Changes in gene expression have been hypothesized to play an important role in the evolution of divergent morphologies. To test this hypothesis in a model system, we examined differences in fruiting body morphology of five filamentous fungi in the Sordariomycetes, culturing them in a common garden environment and profiling genome-wide gene expression at five developmental stages. We reconstructed ancestral gene expression phenotypes, identifying genes with the largest evolved increases in gene expression across development. Conducting knockouts and performing phenotypic analysis in two divergent species typically demonstrated altered fruiting body development in the species that had evolved increased expression. Our evolutionary approach to finding relevant genes proved far more efficient than other gene deletion studies targeting whole genomes or gene families. Combining gene expression measurements with knockout phenotypes facilitated the refinement of Bayesian networks of the genes underlying fruiting body development, regulation of which is one of the least understood processes of multicellular development.

## Author summary

Scientists are just beginning to be able to link comparative developmental biology—which focuses on phenotypes—to comparative genetics (including comparative genomics and transcriptomics). The challenge to doing so has been to coordinate genetic analysis in multiple species, each with distinct genomes, in order to reveal fundamental knowledge of how the modules underlying development evolve and change. In this research, we have brought comparative genetics, developmental biology, genomics, and transcriptomics into a single phylogenetic framework. By using comparative genomic data to identify orthologous genes, comparative transcriptomic data to reconstruct evolutionary changes of gene expression phenotypes across multiple species, and gene knockout technology in more than one species to interrogate function, we have identified a powerful technique to reveal genes whose evolving role has led to developmental and phenotypic differences among species.

## Introduction

In the evolution of complex phenotypes, changes in gene regulation have long been argued to play a greater role than changes in protein function [1–8]. There are many levels at which gene expression level and protein activity can operate or interplay to provide regulation [9–14]. Numerous examples of interspecies variation in the phenotype of gene expression have been identified, many of which can be linked to the evolution of higher, more complex phenotypes [15–29]. Estimation of gene expression phenotypes in ancestral lineages provides a means of identifying putative changes in gene expression that may be key to morphological and adaptive innovation. If complex phenotypes are dependent on changes in gene expression among the key genes that control developmental processes, then changes in morphology should map to changes in gene regulation and expression. Moreover, identification of the changes in the genetic network architecture underlying altered ancestral expression and regulation could open up the “black box” linking genotype to phenotype through development, revealing relevant gene network modularity and identifying the origins of diversity [30].

The recent availability of genome sequences of large numbers of organisms makes this approach feasible, as genomic techniques for measuring gene expression offer the opportunity to comprehensively evaluate the relationship of gene expression to morphology. Identification of ancestral shifts in the level of gene expression using genomic technologies is contingent on the growth of divergent species in a common environment, so that gene expression differences result from genetic rather than environmental effects [31]. Fungi represent a great model for addressing this challenge: they are typically heterotrophic generalists, genome sequences of a large number of fungal species are now available, genomes are relatively small, and genes are well annotated. Additionally, it is easy to perform experimental validation on many fungi via functional approaches. Furthermore, sexual fruiting bodies of fungi offer a degree of multicellular developmental complexity, including tissue differentiation, that represent a good model for more complex multicellular eukaryotes.

**Fig 1:**
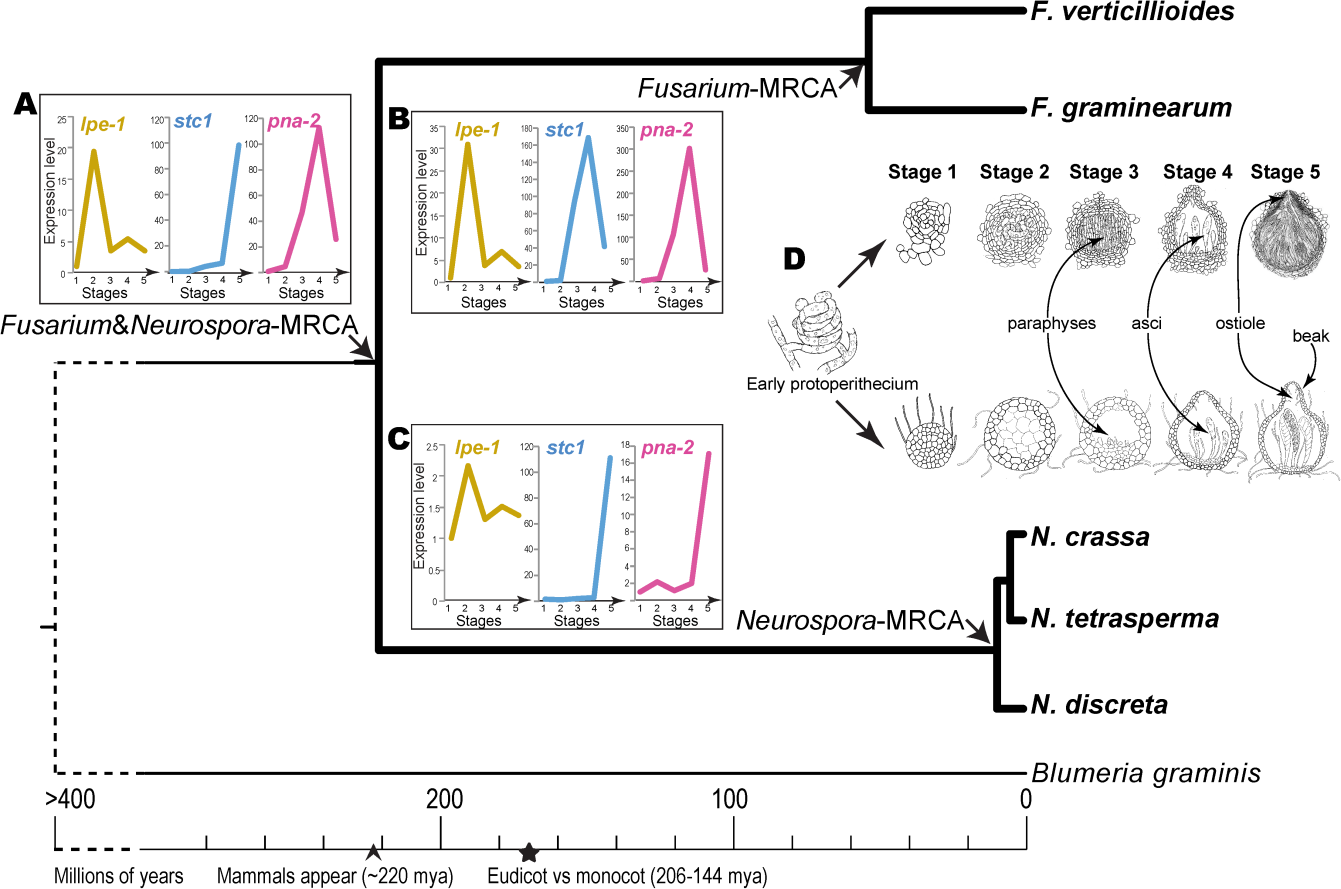
Phylogeny of *Fusarium* and *Neurospora* species, with *Blumeria graminis* as an outgroup, illustrating convergent and divergent evolution in gene expression during morphological development. The phylogeny was estimated from amino acid sequences of the largest (*RPB1*) and the second-largest (*RPB2*) subunits of DNA-dependent RNA polymerase, and the timing was calibrated based on results from Taylor and Berbee [38]. Ancestral expression estimates of *lpe-1*, *stc1*, and *pna-2* genes for the most recent common ancestor (MRCA) of *Fusarium* and *Neurospora* (*Inset A*), for the MRCA of the *Fusarium* species (*Inset B*), and for the MRCA of the *Neurospora* species (*Inset C*) are depicted. Orthologous gene sets across taxa are represented by distinct colors. Stages (1–5) of development (not drawn to scale) are indicated in *Insets* A–C and illustrated in *Inset* D.

The genetics of sexual development in the ascomycetous filamentous fungi has been studied extensively using saprotrophic model organisms such as *Neurospora crassa* [32], *Aspergillus nidulans* [33], and *Sordaria spp*. [34,35] and in the plant pathogen *Fusarium graminearum* [36]. *Neurospora* and *Fusarium* species belong to the orders Sordariales and Hypocreales, respectively, of the Sordariomycetes. They produce complex, flask-shaped fruiting bodies called perithecia (**Fig 1**). The fruiting bodies harbor the meiospores (ascospores) within cellular sacs called asci, which function as cellular cannons to forcibly discharge spores into the air [37]. Asci are formed from ascogenous hyphae, products of mating in self-incompatible species, or self-fertilization in self-compatible species. Thus, fruiting bodies have a role in sexual recombination, but also provide an effective means to local and long-distance dispersal. Ancestors of all species in the genera *Fusarium* and *Neurospora* diverged approximately 200–240 million years ago [38]. However, the stages of fruiting body development in constituent species are morphologically comparable. Fungal fruiting bodies therefore provide an ideal system for the analysis of two questions of central importance to evolutionary biologists: what are the common and divergent developmental sources of homologous phenotypes and how does novelty arise despite the constraints of extant gene interaction networks [39,40].

The ecology of *Neurospora* and *Fusarium spp*. provides an opportunity to relate developmental evolution to their ecology [41, 42,e.g. 43–47]. Perithecia of *Neurospora spp*. and *Fusarium spp*. have retained shared common features such as the ascus structure (**Fig 1**). While transcriptional and morphological divergence among *N*. *crassa*, *N*. *tetrasperma* and *N*. *discreta* and between *F*. *graminearum* and *F*. *verticillioides* have been previously examined [48,49], the two genera have not been compared. The genera differ significantly in morphology. *Fusarium spp*. feature a noticeable stroma: a mass of often darkly pigmented pseudoparenchyma tissue from which the perithecia emerge. In addition, the ontogeny of the paraphyses [a network of sterile hyphae interspersed among the asci and have been shown to assist in spore firing in some species; 50] differs between the two genera in that *Fusarium spp*. form paraphyses that emerge apically, from above the asci [51], whereas in *Neurospora spp*. they emerge laterally and basally [52,53]. Strikingly, *Neurospora* species develop an extended ostiole, or ‘beak’ (**Fig 1**). In *Neurospora* species, perithecia, which are formed in association with fire [54,55], are initiated underneath a layer of plant tissue, use the beak to breach the surface, and eject spores into the air for dispersal. The beaks are phototrophic, and must be fully mature for the spores to discharge. In contrast to *Neurospora*, the perithecia of *Fusarium* lack a beak (**Fig 1**) and form on colonized, senescent host plants (Guenther and Trail, 2005), predominantly small grains. *F*. *graminearum* is reliant on ascospore release for movement from the ground to the flowers of the new crop, from one agricultural field to the next, and across long distances. Thus, the ascospores play a significant role in disease initiation [56,57].

The evolution of new phenotypes requires alteration of the developmental process, which in turn requires alteration of interactions within and among gene modules. The classical means of identifying genes that contribute to phenotypes of interest has been the forward genetic screen, which typically reveals genes by abrogating their biochemical function with mutations [58–70]. In contrast to forward genetic screens, reverse genetics can be guided by genomic assays, such as gene expression profiling. Among genomic assays, gene expression profiling also reveals information regarding the coexpression of genes and gene networks [71–74]. Although reverse genetic approaches based on gene expression data have been used widely to select candidates for functional assays [74–77], assignment of function to new genes has remained challenging because the high number of candidate loci [77–82]. Furthermore, reverse genetic approaches provide contextual clues to gene interactions and modules that are relevant to phenotypes and their evolution.

To guide our investigation of genes and gene modules relevant to the process of fruiting body development, we focused on five previously characterized stages of perithecium development. Comprehensive profiling of gene expression across these stages has been conducted in three species of *Neurospora*—*N*. *crassa*, *N*. *discreta*, and *N*. *tetrasperma* [48], and in two species of *Fusarium—F*. *graminearum* and *F*. *verticilloides* [49,83]. All species were cultured in a common medium to accurately identify genetically encoded expression differences between orthologs essential to morphological divergence. Continuous ancestral character estimation facilitated identification of the largest changes in gene expression specific to common stages of perithecium development in the most recent common ancestors of the *Neurospora* species and of the *Fusarium* species, resulting in a phylogenetically explicit, comparative approach enabling the identification of developmental mechanisms that can be associated with phenotypic change [39]. Functional analysis of identified genes was performed in *N*. *crassa* and *F*. *graminearum*. To demonstrate a potential basis of these traits in gene interactions, we generated Bayesian networks computed on the basis of gene expression correlations. Comparison of Bayesian networks constructed across nodes of the phylogenetic tree provided hypotheses for the evolution of regulatory modules [30]. Finally, we assessed whether theoretical predictions based on the evolution of gene expression that identify genes playing roles in the evolution of an important trait can be evaluated by experimental analysis of function to reveal the relation between gene, development, and phenotype.

## Results

### Developmental sequence

In culture, sexual development of *F*. *graminearum*, which is self-compatible, and *N*. *crassa*, which is self-incompatible, developed in accordance with developmental stages previously described [82]: generating four “tissue types” (ascogenous hyphae, perithecial wall, paraphyses, asci) and mature spores. Each stage began with the appearance of a tissue type and ended at the initiation of a subsequent tissue type. Stage 1 was initiated as the ascogenous hyphae began to develop from the perithecium initials (protoperithecia; **Fig 1**); Stage 2 was initiated as the perithecial wall differentiated; Stage 3 began as the paraphyses started to form; Stage 4 began as the asci emerged; Stage 5 began as the ascospores differentiated, and ended with mature spores in a mature perithecium. In Stage 5, a beak was fully formed at the apex of the perithecium in *N*. *crassa*, but not in *F*. *graminearum*. Squash-mounts of a perithecium at the end of Stage 5 in both species (approximately 144 h after initiation of sexual development) revealed the inside of the perithecium, containing mature asci with ascospores (**S1 Fig** and **S2 Fig**). Across the entire time course of perithecial development, protoperithecia darkened from grayish to yellowish gray in the *Neurospora spp*., and continued to darken to almost black in maturing perithecia [48]. In contrast, the protoperithecia of *F*. *graminearum* were carmine, deepening to purplish-black as the perithecia matured [51].

### Ortholog predictions and comparative gene expression

**Fig 2:**
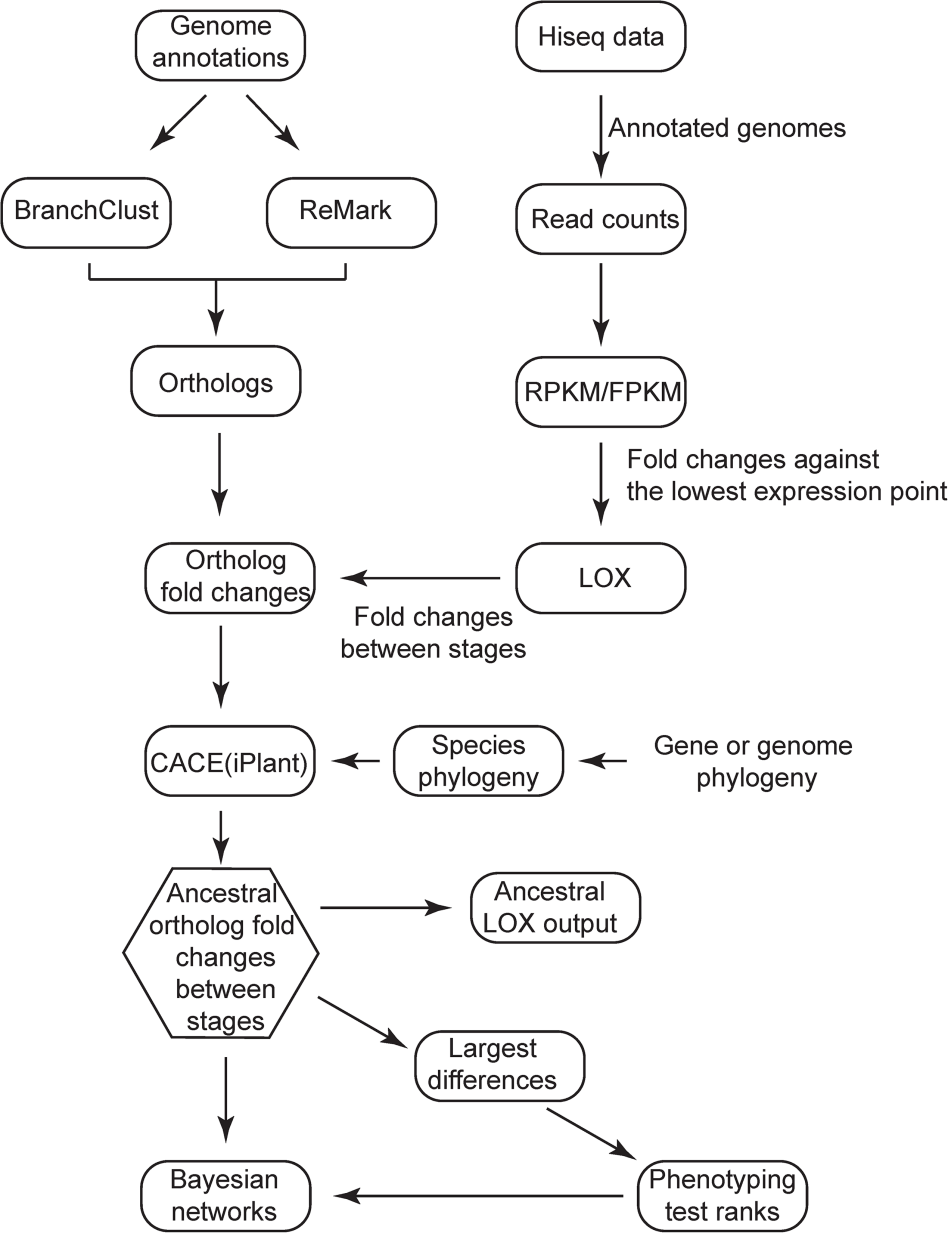
Flow chart for ortholog predictions, sequence data, phylogeny, and phenotyping.

To conduct comparisons of gene expression across species (**Fig 2**), we analyzed the predicted gene models for all *Neurospora* and *Fusarium* species (**Fig 1**) using ReMark [84], which operates using the BLAST algorithm [85], and BranchClust [86], which conducts a gene tree based approach to ortholog identification. Doing so, we identified 4,668 single copy orthologous gene sets with representation in all five species (**S1 Table**). Slightly more ortholog sets were identified by BranchClust (10 additional sets) than were identified by ReMark, and all these 10 sets were further verified with a manual reciprocal best BLAST with amino acid sequences criterion among the five genomes included in the analyses. Approximately 2000 gene sets from the ReMark analysis comprised multicopy homologs within the five species. Due to the complex analysis of gain and loss that would be required, these gene sets were excluded from further analysis in this study. Gene expression levels were measured during perithecium development in *Neurospora spp*. and *Fusarium spp*. in previous studies for all genes identified within the orthologous sets. Of the 4668 single copy orthologs, one or more reads were identified for 4490 orthologs for all five species across all sample points for all stages. Consistent with previous separate analyses of *Neurospora* and *Fusarium* gene expression, fundamental cellular processes exhibited common patterns among sister species. For instance, across all five species, mitochondrial ribosomal gene expression was upregulated during initial stages of perithecial development, and expression of nuclear ribosomal genes peaked during formation of the asci.

### Generation of a prioritized list of genes whose expression evolved

**Fig 3:**
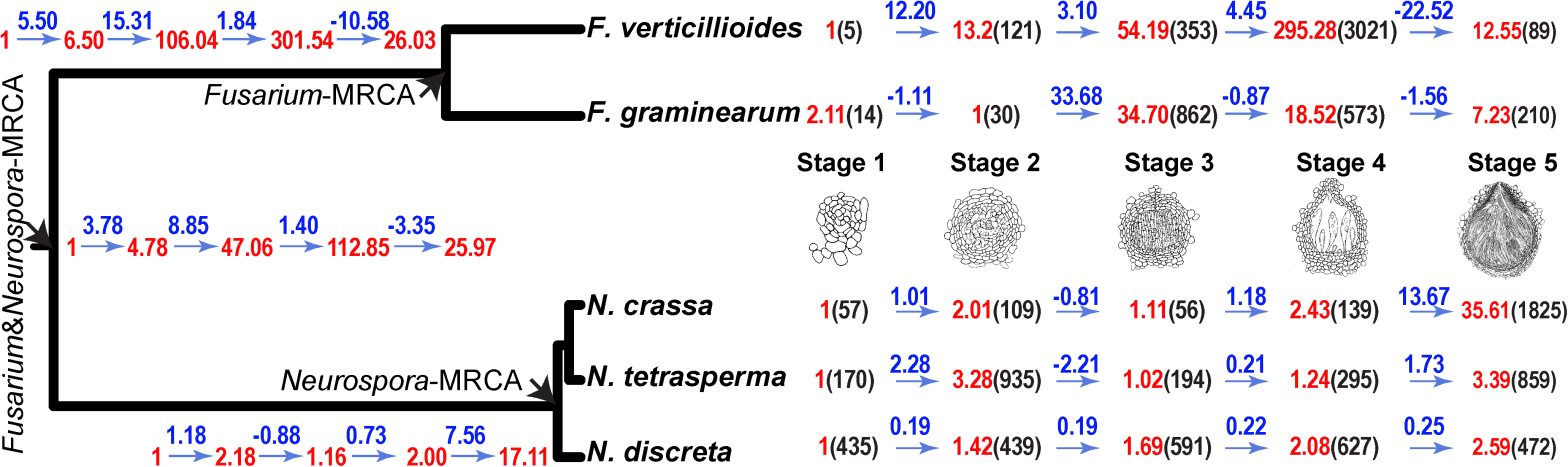
Example of fold increases and decreases in expression between developmental stages (blue, based on (*X*_*t+1*_*-X*_*t*_) / *min* [*X*_*t*_, *X*_*t+1*_], where *X* is the expression level and *t* is the stage), the continuous ancestral state estimations of the fold increases and decreases between stages for nodes of interest (also blue), the corresponding estimates of stage-to-stage relative expression across stages normalized to the stage of lowest expression (red), and the raw (un-normalized) sequencing counts (black, in parentheses). Numerical values depicted are for the orthologs of *pna-2*, for which estimated gene expression in the most recent common ancestor (MRCA) of the *Neurospora* species increases markedly between stages 4 and 5, whereas estimated gene expression in the ancestor of *Fusarium* drops markedly between stages 4 and 5. In this research, we found that a knockout of *pna-2* exhibits a sexual development phenotype starting at stage 4 in both *N*. *crassa* and *F*. *graminearum*.

We hypothesized that genes whose expression increased substantially in one lineage during divergence of *Fusarium* and *Neurospora* (e.g. **Fig 3**) are important to the evolution of morphological differences between these genera.

Thus, we ranked genes in each lineage (*Fusarium* and *Neurospora*) by differences in expression between the most recent common ancestor of the two *Fusarium* species (MRCA-F; **Fig 1**) and the MRCA of the three *Neurospora* species (MRCA-N; **Fig 1**), providing a candidate gene knockout prioritization list, identifying those that have putatively evolved function during perithecial development and therefore might be crucial for the successful formation of perithecia. Using continuous ancestral state inference based on the phylogeny, we estimated the developmental time course of gene expression for each ortholog (**S1 Table**) of the most recent common ancestor of each subclade (ancestral nodes; **Fig 1**and **S2 Table**). Our analysis of ancestral levels of gene expression identified genes that underwent a recent shift in expression, in particular shifts that occurred along the shared ancestral branch leading to MRCA-N and MRCA-F.

### Functional analysis of ranked orthologs

We hypothesized that knockouts of genes that recently underwent an increase in expression would exhibit phenotypes during sexual development, in particular at the stages of morphological divergence where the increase occurs. Therefore, we scored the stage of each knockout phenotype obtained, defined as the first stage (**Fig 1**) in which a definitive aberrant phenotype was observed. For ease of discussion of genes that were not already named in either species, we assigned names based on the knockout phenotype in either or both species (**Table 1**).

**Table 1:** Phenotypes of strains with orthologous genes knocked out in both species.

Gene	*F*. *graminearum*	Knockout phenotype *F*. *graminearum*	*N*. *crassa*	Knockout phenotype *N*. *crassa*
	FGSG_00426	Wild type	NCU09140	Wild type
*div-12*	FGSG_00565	Few perithecia, no spores	NCU02496	Wild type
*rel-5*	FGSG_01108	Stage 5	NCU03098	Wild type
*asy-1*	FGSG_02102	Asynchronous	NCU07748	Wild type
*pdv-1*	FGSG_02751	Wild type	NCU08856	Stage 3
*sdi-4*	FGSG_03028	Stage 1	NCU04197	Wild type
	FGSG_03813	Wild type	NCU09775	Wild type
*rel-1*	FGSG_04001	Stage 5	NCU04520	Wild type
	FGSG_04180	Wild type	NCU07924	Stage 1
*stc1*	FGSG_04417	No asci, mature wall	NCU01496	Short beak
*pna-2*	FGSG_04997	Stage 5	NCU06316	Short beak
*lpe-2*	FGSG_05166	No asci, few perithecia	NCU03490	Wild type
*asy-2*	FGSG_05652	Asynchronous developing perithecia; no cirrhi; reduced firing	NCU06985	Wild type
*vad-1*	FGSG_06651	Few perithecia, delayed development	NCU00329	Wild type
*asl-1*	FGSG_07111	No asci, perithecial wall is fully developed	NCU07508	Perithecia with beak, no asci or ascospores
	FGSG_07376	Wild type	NCU01374	Wild type
*lpe-1*	FGSG_07478	Limited numbers of perithecia, delayed development	NCU01451	Abnormal ostioles, no ascospores
*pls1*	FGSG_08695	Increased number perithecia	NCU07432	Wild type
	FGSG_09006	Wild type	NCU01140	Wild type
*sbk-2*	FGSG_09475	Wild type	NCU09788	Small beaks
*rel-2*	FGSG_10094	No cirrhi, reduced firing	NCU01009	Wild type
	FGSG_12680	Wild type	NCU07009	Wild type
*pdv-1*	FGSG_13162	Small perithecia, no asci	NCU06930	Wild type
	FGSG_16340	Arrested at protoperithecia	NCU00552	Lack of carotenoid pigments
*rel-4*	FGSG_17494	No cirrhi, reduced firing	NCU02908	Wild type
*asl-3*	FGSG_17499	No asci, perithecial wall is fully developed	NCU06136	Wild type

### Analysis of ranked orthologs in *F*. *graminearum*

In *F*. *graminearum*, knockouts were completed on 23 genes from the ranked list (**S3 Table**) among the top 10% of all predicted orthologs, all of which were estimated by our ancestral state analysis to have evolved markedly increased expression at one or more stages of sexual development. Of these genes, 17 (74%) exhibited knockout phenotypes affecting sexual development (**S1 Fig** and **S3 Table**). In early development, wild type *F*. *graminearum* produces a minimal stroma, pigmented red, which replaced the hyphal mat on the surface of the agar and from which the perithecia arise. Knockout strains of nine genes were arrested at developmental Stage 1. These included those arrested in development at the protoperithecia stage [FGSG_03028 (*sdi-1*), FGSG_16340; **S1 Fig**] and those with limited numbers of perithecia, where many protoperithecia did not develop further [FGSG_00565 (*div-12*); 5166 (*lpe-2*; *l*imited *pe*rithecia); 6651 (*vad-1*); 7478 (*lpe-1*)], and those where many more protoperithecia matured into perithecia than the wild type [FGSG_08695 (*pls-1*); discussed below]. Also included were knockouts of *asy-1* and *asy-2* (for *asy*nchronous FGSG_02102 and FGSG_05652, respectively), which exhibited asynchronous development of perithecia across the surface of the agar, with the perithecia maturing normally, but Δ*asy-2* produced no cirrhi (see below) and had reduced firing of spores (discussed below). There was not a striking shared pattern of expression among these genes, although the majority expressed at their highest levels during earlier stages.

Ascospores of *Neurospora* and *Fusarium* species are forcibly fired from asci, however, they can also be exuded from the perithecial pore *en masse* as a cirrhus, which usually occurs with spores remaining inside an aging perithecium, or under suboptimal firing conditions. Five gene knockouts in *F*. *graminearum*, Δ*rel-1* (FGSG_04001; *rel* for spore *rel*ease), Δ*rel-2* (10094), Δ*rel-4* (17494, formerly 10943), Δ*rel-5* (1108), and Δ*asy-2* affected spore release (Stage 5), but in slightly different ways. Knockouts of genes *rel-1*, *rel-2*, *rel-4*, and *asy-2* developed mature perithecia with reduced spore discharge, and produced no cirrhi. Knockouts of *rel-5* produced cirrhi several days earlier than the wild type cultures, and knockouts of *pls-1* exhibited an increased number of perithecia and early spore ejection. The *rel* genes peaked in expression in Stages 3–5, when the asci are forming, whereas *pls1* peaked at Stage 1, supporting the inclusion of this knockout as a Stage 1 mutant, as the knockout phenotype of increased numbers of perithecia is consistent with its activity at a very early stage.

Knockouts of *pdv-2* (FGSG_13162) were arrested in development at Stage 2, forming very small perithecia. Knockouts of three genes were arrested in development of perithecia at Stage 3 (**Fig 3**and **S1 Fig**).The asci also did not develop in knockout strains of *asl-1* (*as*cus-*l*ess; FGSG_07111) and *lpe-2*, and the perithecial wall developed to the size characteristic of wild-type perithecia at this stage. In contrast, the perithecial wall formed by knockout strains of *stc1* (homolog of *s*iRNA *t*o *c*hromatin in fission yeast, FGSG_04417) and *asl-3* (FGSG_17499, formerly 13889) matured to full size and appearance, but no asci formed inside. Knockout strains for six genes (FGSG_00426, 03813, 04180, 07376, 9006, 12680) exhibited wild-type morphology.

To show that the phenotypes resulted from the insertion of the genetic marker gene *hph1*, encoding hygromycin phosphotransferase for resistance to the antimicrobial hygromycin, into the target gene, colonies from single ascospores were isolated from recombinant cirrhi of crosses between strain PH-1-55 (*nit-3* mutant) and knockout strains of four genes (*asy-1*, *rel-2*, *stc1*, *pdv-2*). Segregation ratios supported the cosegregation of hygromycin resistance and the gene knockout phenotypes (**S4 Table**). For analysis of Δ*asy-1*, the NIT^−^ phenotype, hygromycin resistance and the knockout phenotype co-segregated with Δ*asy-1*, due to the colocation of the *nit-3* locus with *asy-1* on chromosome 1. PCR analysis verified the integration of *hph1* and the loss of the target genes at the integration sites.

### Analysis of ranked orthologs in *N*. *crassa*

**Fig 4:**
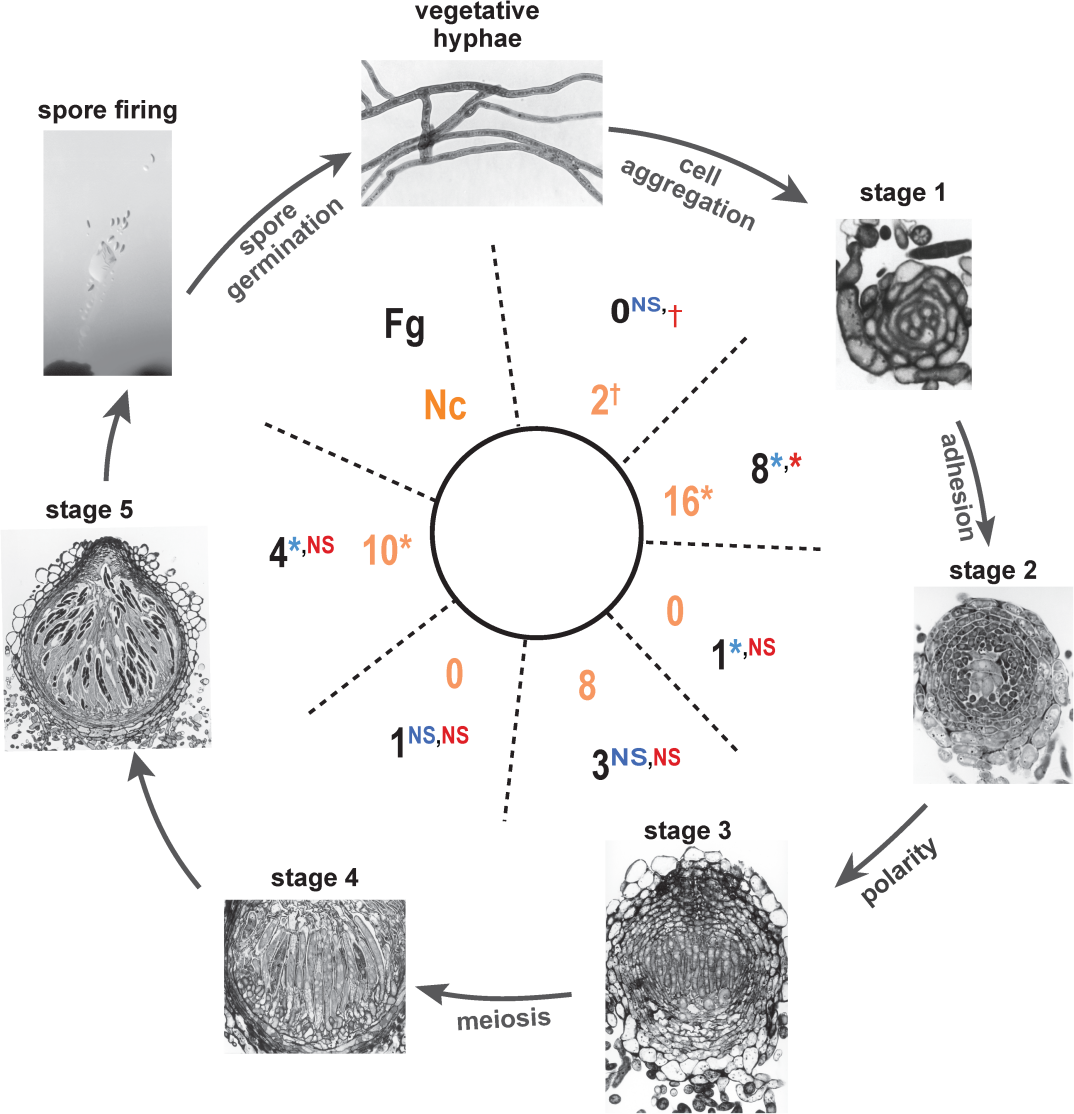
Distribution of phenotypes for knockout strains across stages of development in *F*. *graminearum* and *N*. *crassa*. For each stage, numbers of genes exhibiting knockout mutant phenotypes are reported in the center (outside in black, *F*. *graminearum*; inside in orange, *N*. *crassa*). These numbers were compared to the distribution reported in three previous systematic studies—outside left (blue), analyzing function of the protein kinase genes [97]; outside right (red), functional analysis of transcription factor genes in *F*. *graminearum* [96]; and inside (tan), functional analysis of genes across the genome in *N*. *crassa [87,88]*—yielding significantly higher representation of phenotypes per knockout (*), significantly lower representation of phenotypes (†), or no statistically significant difference (NS). Revision of figure reproduced with permission from: Sex and Fruiting in *Fusarium*. F. Trail. *In* Fusarium: Genomics, Molecular and Cellular Biology, Chapter 2, D.W. Brown and R. H. Proctor, Eds., Caister Academic Press pp. 11–30 [36].

Of 196 top-ranked genes in *N*. *crassa* (**S5 Table**) that were available from the genome-wide knockout project [87,88], mutant phenotypes in sexual development were observed for knockouts of 41 genes (**S5 Table**), with 36 of these exhibiting phenotypes affecting discrete stages of perithecial development (**Fig 4**and **S2 Fig**). Similar to our results with the knockout phenotypes in *F*. *graminearum*, many knockout phenotypes in *N*. *crassa* were arrested during early perithecium development or were developmentally perturbed in late perithecial development (**Fig 4**). Knockouts of two genes, *sah-1* (NCU04179) and *s*porulation *r*elated *g*ene *srg* (NCU09742), were only available from one of the two mating types and produced perithecia only in one parent when crossed with the wild type strain—that is, perithecia only developed when the wild type strain was the perithecial parent in a cross with the knockout strain for *sah-1* and when the knockout strain for *srg* was the perithecial parent in a cross with the wild type strain. These results suggest that these genes function in the initiation of perithecia before Stage 1, possibly during mating. Based on its late function in spore release in *F*. *graminearum* as assessed by knockout phenotyping, this developmental timing for the ortholog in *N*. *crassa* is earlier than its expected time of action.

Knockouts of 16 genes in *N*. *crassa* yielded phenotypes in protoperithecial morphology and development (Stage 1). The knockout strain for NCU07817 (*ncw-3*) produced protoperithecia arrested at a very early stage. The strain with NCU03247 knocked out produced protoperithecia at least 48 h earlier than the wild type, then proceeded to develop mature, wild type perithecia (**S2 Fig**). Among the other fourteen genes, there were slight differences in morphology, number, and size of protoperithecia (**S2 Fig**). Of all 16 genes, 13 exhibited higher expression (10 statistically significantly) at the protoperithecia stage compared with later stages, and two genes—NCU03121 and 04713—were most highly expressed in protoperithecial samples compared to subsequent stages. The remaining three genes (NCU03492, 03247, and 07449) exhibited an expression pattern that was common across the genome: increasing expression across the sampled stages during sexual development in *N*. *crassa*. For the majority of the genes identified as having important roles in development subsequent to protoperithecia, expression spiked during the stage at which the knockout phenotype appeared.

Of the 18 genes in *N*. *crassa* with knockout phenotypes after Stage 1, eight yielded knockout phenotypes in Stage 3. Knockout strains of six of these genes produced neither asci nor ascospores (NCU01451, 01460, 01496, 01760, 06316, 07508, and 08856). The *mat-a* knockout strain of NCU08428 produced slowed perithecium development. Knockouts of NCU07508 (hollow, *hol*) produced a phenotype unique to this study, a fully normal-shaped wall and beak, but no asci, perhaps similar to the similar phenotype of *stc1* and FGSG_17499 in *F*. *graminearum* in forming a fully mature wall, but no asci. Because beak development in *N*. *crassa* occurs during Stage 5, full beak development is ordinarily assumed to be “downstream” of the first formation of asci and ascospores. Previous studies have suggested beak morphology is genetically controlled by the female (protoperithecial) parent in *N*. *crassa* [89,90]. While no knockouts in *N*. *crassa* produced unusual morphologies in ascospores or asci (Stage 4), knockouts for ten genes (NCU00317, 01120, 01134, 01640, 02879, 03938, 05858, 07621, 09788, and 09915) exhibited phenotypes at Stage 5, affecting beak development and spore release. Compared with wild-type beaks with lengths of 0.16–0.20 mm, these mutants showed significantly shorter beaks (**S3 Fig**), with the largest ranging from 0.11–0.17 mm (Δ*sbk-1* NCU02879) and the smallest ranging from 0.03–0.09 mm (*fsd-1* NCU09915). Knockouts of *aod-5* (NCU03938) produced some perithecia arrested early in development and others that form a short beak and do not release spores. Knockouts of *tzn-1* (NCU07621) showed greatly reduced spore release probably due to the very reduced beak characteristic of these strains. Of the 10 genes exhibiting mutant phenotypes in beak development and morphology, all except *rpn-4* (NCU01640) exhibited significant up-regulation after Stage 3. In summary, a large number of beak mutants were recovered from our study, with many of the genes upregulated late in development, when beaks form.

Of the 196 gene knockouts assessed in *N*. *crassa*, phenotypes of 110 gene knockouts were assessed as homozygous crosses of knockouts, whereas 86 were available only in one mating type and were assessed in a heterologous cross with the wild-type strain of the opposite mating type. Mutant phenotypes in sexual development were observed for knockouts of 41 genes—27 homozygous and 14 heterozygous. Thus, homozygous knockouts yielded a higher percentage of phenotypes (25% vs. 16%, a difference that is not quite statistically significant by a one-tailed Fisher Exact Test, *P* = 0.11). Six *N*. *crassa* knockouts failed to produce perithecia in crosses with wild type strains. Five *N*. *crassa* knockouts produced too few ascospores for a segregation test. The observed knockout phenotypes in sexual development in *N*. *crassa* for the other 30 knockouts fully segregated with Hyg^r^ in the progeny of crosses to wild type *N*. *crassa*, thus demonstrating that the deletion was responsible for the loss of gene activity. Of the 41 knockouts in *N*. *crassa* that had knockout phenotypes in sexual development, 27 were homozygous and 14 were heterozygous, whereas all of the deletion mutants obtained in *F*. *graminearum* were homozygotes. Although this disparity between the frequency of observation of knockout phenotypes in homozygous versus heterozygous crosses of *N*. *crassa* is not statistically significant, it implies that some of the difference between the percentage of knockouts exhibiting phenotypes in *F*. *graminearum* (74%) and the percentage of knockouts exhibiting phenotypes in *N*. *crassa* (22%) may be attributable to the substantial fraction of knockout mutants in *N*. *crassa* that were examined only as heterozygous deletions.

We subjected the 43 genes in *N*. *crassa* that exhibited knockout phenotypes in perithecial development to manual BLAST analyses against the *S*. *cerevisiae* (R64-1-1) reference genome, yielding 10 identifiable orthologs in *S*. *cereviseae* (**S6 Table**). Of 15 genes that were classified as “hypothetical proteins” in *N*. *crassa*, one (NCU09443) is an apparent ortholog of the yeast *ste6*, which functions in the export of the alpha-factor pheromone in yeast [91]. The knockout of NCU09443 was arrested in development at the protoperithecial stage. Interestingly, knockouts of *tzn-1* (NCU07621) and *sbk-1* (NCU02879) exhibited phenotypes of short, small beaks in *N*. *crassa*. BLAST analysis against *S*. *cerevisiae* indicates that they may function as zinc/iron transporters in yeast [ZRT1 and ZRT2; 92, 93]. Finally, two *N*. *crassa* genes (NCU01120 and NCU09915) were best BLAST hits to meiosis-specific genes in yeast *spo11* [94] and *ndt80* [95]. Knockouts of these genes in *N*. *crassa* aborted sexual reproduction, producing no ascospores or producing abnormal sexual spores.

### Functional analysis of ranked ortholog Pairs in *N*. *crassa* and *F*. *graminearum*

To ascertain whether knockouts of *N*. *crassa* genes would exhibit similar or different knockout phenotypes compared to their orthologs in *F*. *graminearum*, we assessed the phenotypes of knockouts of the *N*. *crassa* orthologs of the 23 highly ranked genes that were knocked out in *F*. *graminearum* (**Table 1**). Of the 23 gene knockouts of orthologs in *N*. *crassa*, 17 exhibited a wild type phenotype. Of the six genes that exhibited wild type knockout morphology in *F*. *graminearum*, only one exhibited a knockout phenotype (the knockout of the *N*. *crassa* ortholog of FGSG_4180 (NCU7924) was arrested at protoperithecium development). To further compare the evolution of gene function, we knocked out two additional genes in *F*. *graminearum* whose orthologs had exhibited knockout phenotypes in *N*. *crassa* (*pdv-1*, *sbk-2*) and both had wild type knockout phenotypes in *F*. *graminearum*. In summary, there were no exact matches of knockout phenotypes in orthologs between *N*. *crassa* and *F*. *graminearum*. Moreover, there were many cases where the knockout of the ortholog in the other species yielded no observable knockout phenotype. However, the peaks in expression corresponded in many cases to the appearance of arrested development in the knockouts; some genes exhibited bimodal peaks in expression with the knockout phenotype corresponding to the earlier peak.

### Rates of knockout phenotype discovery based on evolved increases of gene expression

We predicted that in using the estimated evolved differences in gene expression to guide our candidacy for gene knockouts, we would identify significant numbers of genes involved in sexual development, compared to knockout analyses of the whole genome, or of functionally-defined subsets. In *N*. *crassa*, in nearly one out of four genes (44 out of 196), we observed knockout phenotypes in sexual development, a higher ratio than that produced by the entire set of strains from the whole-genome knockout project [87,88] in which 235 of more than 2000 genes whose knockouts were phenotyped were reported to be arrested during sexual development (two-tailed Fisher Exact Test *P* < 0.01). In *F*. *graminearum*, our study yielded 17 of 23 genes that produced knockout phenotypes in sexual development, a significantly higher proportion of genes compared to an analysis of genes within the kinome in which 25 of 96 genes yielded knockout phenotypes in sexual development (*P* < 0.0001), and a significantly higher proportion than in a knockout study of 657 transcription factors [96] that identified 75 genes with knockout phenotypes in sexual development (*P* < 0.0001). On a per-knockout basis, our approach identified genes functioning in Stage 1 and later stages of sexual development equivalently or at a statistically significantly better rate than previous studies (**Fig 4**). It identified genes important to the initiation of sexual development (prior to Stage 1) at equivalent or significantly lower rates than previous studies (**Fig 4**).

Consistent with our motivating hypothesis that large increases in expression correspond with gains of developmental function underlying phenotypes of interest, we observed that the stage of greatest comparative increase in gene expression was frequently coincident with the stage of appearance of the knockout phenotype. Examination of the ancestral expression analysis for MRCA-N in comparison to the MRCA-FN (**S3 Table**) revealed that of the 31 genes from the ranked list that exhibited a phenotype that could be staged in sexual development, 16 exhibited knockout phenotypes ascribed to the same stage as the largest upregulatory differences between stages in MRCA-N compared to MRCA-FN. An examination of the *Fusarium* data (**S5 Table**), showed similarly that for 10 of 17 genes with knockout phenotypes, the largest upregulatory differences between MRCA-F compared to MRCA-FN occurred in the stage where the knockout phenotype first appeared.

To constitute a comparison group for the rate of knockouts with phenotypes in perithecium development expected, we examined the rates of knockouts with phenotypes in perithecium development in the *N*. *crassa* program project systematic knockout study. In this comprehensive study, 7.3% of knockouts exhibited a phenotype in sexual development. Of these knockouts that are in our ortholog matrix, 3.7% of the knockouts exhibited a phenotype in sexual development as identified in the KO1 project. In the present study, 18.3% of knockouts in *N*. *crassa* exhibited a phenotype in sexual development, demonstrating the power of our approach. No undirected systematic knockout study has been performed in *F*. *graminearum*.

We assessed whether other criteria historically used to select genes for functional analysis yielded sets of genes whose rankings in our prioritization were similarly high. A review by Trail [36] summarized the characterized knockouts in *F*. *graminearum*, indicating the stage at which development was arrested. Of 103 genes compiled from studies on single genes or small sets of genes, plus the kinase and transcription factor knockout projects [96–98]; 61 had orthologs in *N*. *crassa* and thus were among the 4431 common orthologs considered in our study. Only nine of these orthologous genes fell within the top 10% of ranked genes for *F*. *graminearum*. This proportion (9/391) within our ranked list that were identified by other approaches is not statistically different from the expectation (61/4431; Fisher’s exact *P* = 0.22). Thus, our approach to identification of genes involved in sexual development yielded a high hit rate, and identified genes independently compared to previous approaches.

### Bayesian network predictions for early and late perithecial development

**Fig 5:**
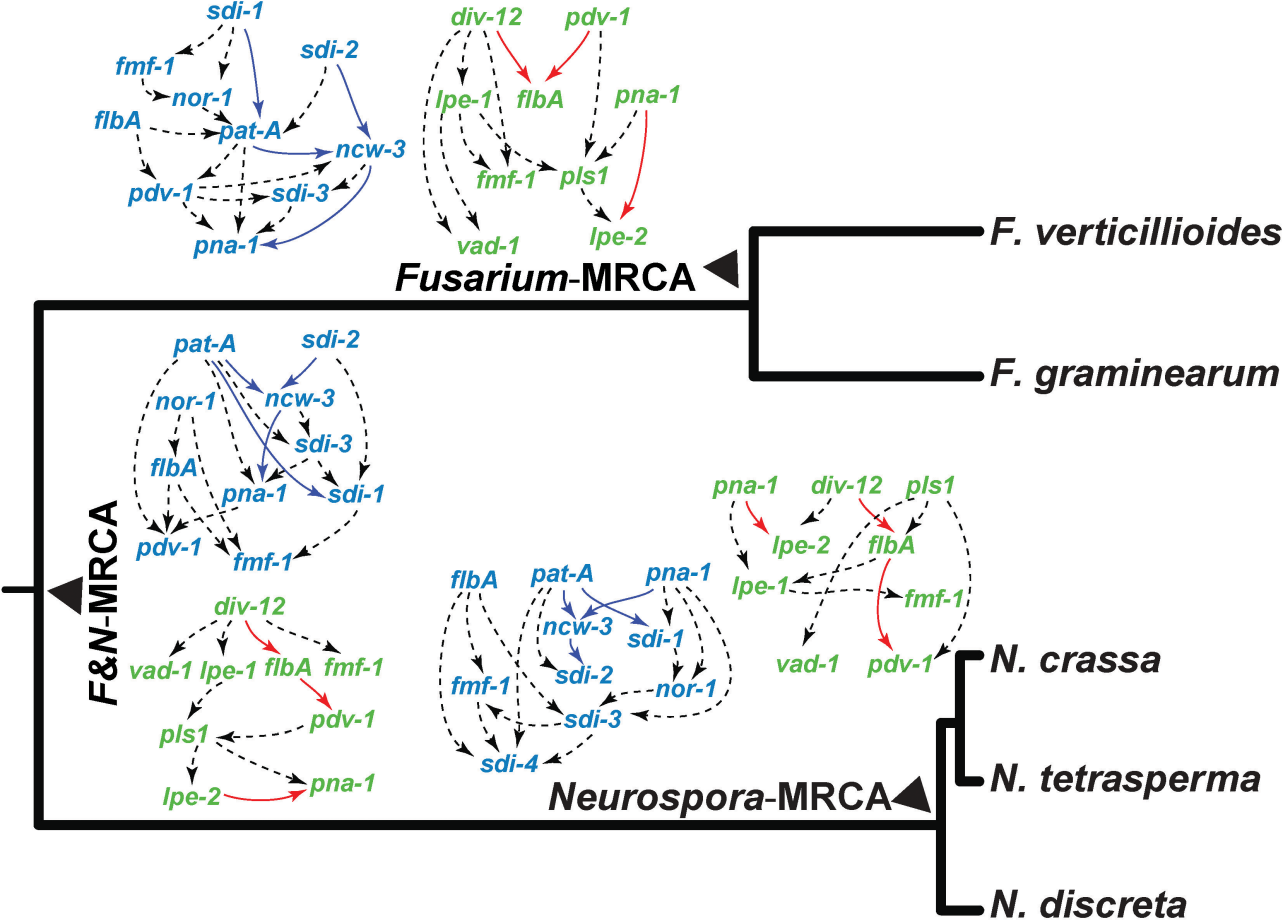
Gene interaction networks underlying early perithecial development in *N*. *crassa* (left, blue) and *F*. *graminearum* (right, green), represented by directed acyclic graphical models of selected genes (S7 Table), displayed in proximity to the cognate ancestor in the phylogeny of *Neurospora* and *Fusarium* species. Genes within networks were suggested by assembling genes with common knockout phenotypes observed within each species. Networks were inferred from the expression level changes measured between all equivalent stages of development in the three most recent common ancestors of these *Neurospora* and *Fusarium* species. Arrows indicate an inferred causal dependency of the two genes they connect. Edges are depicted by dashed black arrows, unless they are present within all three putative ancestral nodes regardless of orientation, in which case they are depicted in blue within the *Neurospora*-specific network, or pink within the *Fusarium*-specific network.

Bayesian networks were generated to compare the potential interactions among genes between *N*. *crassa* and *F*. *graminearum* and to provide models for how gene interaction networks change over evolution. We examined interactions in early and late fruiting body development, focusing on genes involved in beak development in *N*. *crassa* and spore release in *F*. *graminearum*, as well as genes involved in early protoperithecia and perithecial development in both species (**Table 1**). We included three previously characterized genes whose knockouts formed perithecia without asci and ascospores in both *N*. *crassa* and *F*. *graminearum*: sexual development regulatory genes *flbA* and *fmf-1* and transcription factor gene *pna-1* [87,88] to comprehensively analyze the evolution of the early sexual development network (**Fig 5**).

**Fig 6:**
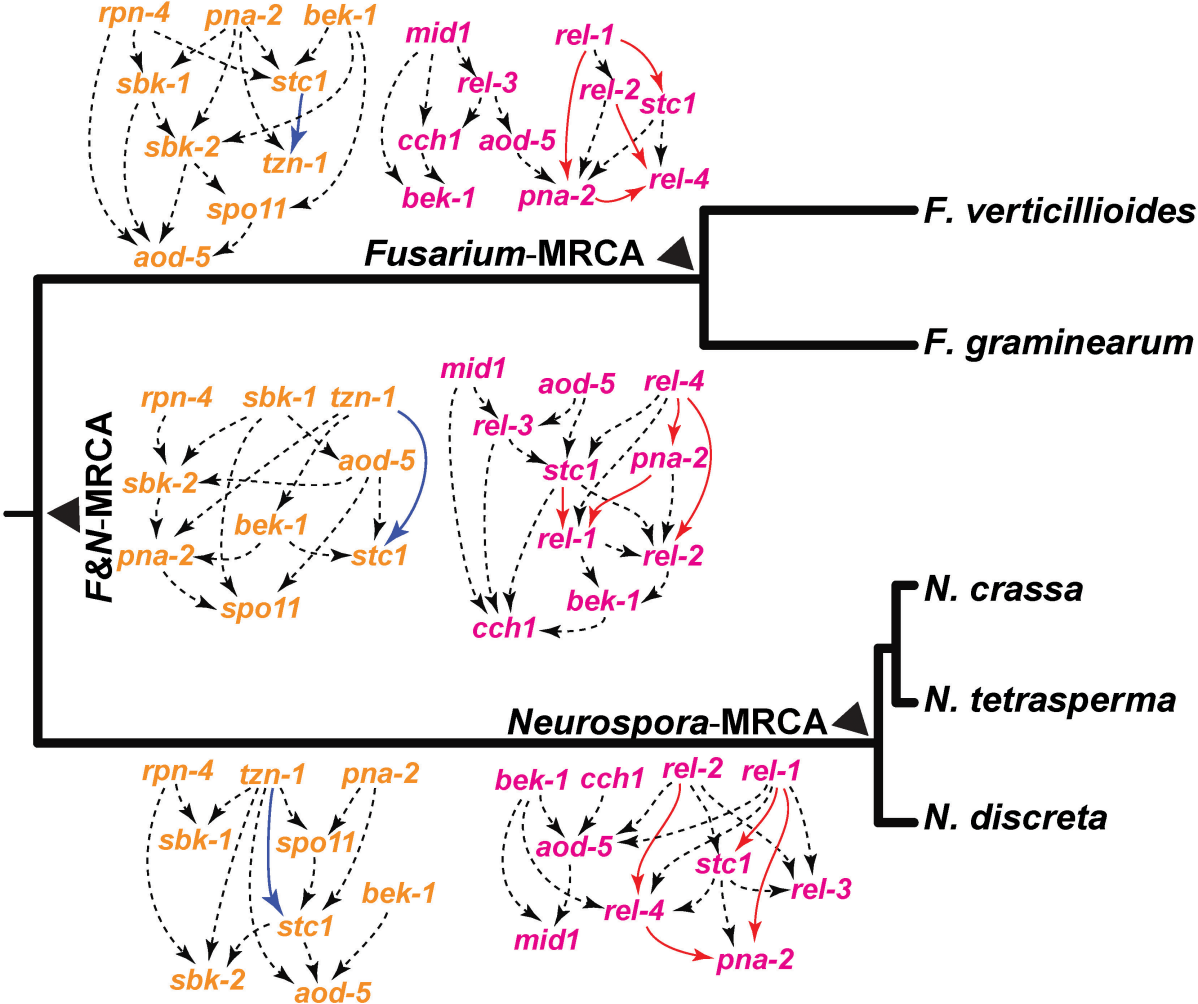
Gene interaction networks underlying beak formation in *N*. *crassa* (left, orange), and ascospore release in *F*. *graminearum* (right, pink), represented by directed acyclic graphical model of selected genes (S7 Table), displayed in proximity to the cognate ancestor in the phylogeny of *Neurospora* and *Fusarium* species. Genes within networks were suggested by assembling genes with common knockout phenotypes observed within each species. Networks were inferred from the expression level changes measured between all equivalent stages of development in the three most recent common ancestors of these *Neurospora* and *Fusarium* species. Arrows indicate an inferred causal dependency of the two genes they connect. Edges are depicted by dashed black arrows, unless the edge is present within all three putative ancestral nodes regardless of orientation, in which case they are depicted in blue within the *Neurospora*-specific network, or red within the *Fusarium*-specific network.

In *N*. *crassa*, these regulators and seven other genes that we identified exhibited a common knockout phenotype of arrest at the protoperithecium stage or arrest in early perithecial development. Similarly, we inferred Bayesian networks for these common regulators and five genes identified in *F*. *graminearum* that exhibited knockout phenotypes affecting protoperithecium development (**Fig 5**). Network connections among the genes with knockout phenotypes in early development were divergent between inferred ancestral species in the phylogeny, in accordance with observed diverse expression profiles across species, and indicating evolving roles in the regulation of development. In particular, genes functioning in the initiation of protoperithecia appear to interact differently in *N*. *crassa* and *F*. *graminearum*. A close association between *pdv-1*, *flbA*, and *fmf-1* persists in all networks predicted for genes involved in early sexual development, and in *N*. *crassa*, Δ*pdv-1* was arrested at an early stage, during initial perithecial development. Knockouts of transcription factor genes *pna-1*, *flbA*, and *fmf-1* arrested during early sexual development in both *N*. *crassa* and *F*. *graminearum*. Despite the persistent function of *pna-1* in sexual development in *N*. *crassa* and *F*. *graminearum* and the maintenance of this ortholog in *N*. *tetrasperma* (NtLA_12702), orthologs of *pna-1* appear to have been lost in *N*. *discreta* and *F*. *verticillioides*. The conditional dependencies of gene expression and therefore the inferred network interactions of *pna-1* also shift, suggesting that its role within a consistent developmental process is changing over time. For genes showing knockout phenotypes in late perithecial development, including beak formation in *N*. *crassa* and spore release in *F*. *graminearum*, expression was prominently up-regulated during late perithecial development in *N*. *crassa* and *F*. *graminearum* respectively. Networks were predicted separately for orthologs of beak-related genes in *N*. *crassa* and spore release genes in *F*. *graminearum* (**Fig 6**) and included two genes, *aod-5* and *bek-1*, which were previously found to exhibit knockout phenotypes in ascus and ascospore development in both species [96,99]. In general, the conditional dependencies of gene expression of these genes (and genes with known meiotic function *spo11* and *stc1*) shift among species and among inferred ancestors, suggesting that their roles in both developmental processes are complex and are changing over evolution.

## Discussion

We have demonstrated that evolved changes in the level of gene expression provide guidance toward recent shifts in gene function during perithecium development. The magnitude of evolved changes in expression of orthologous genes in five fungal species and their putative ancestors allowed us to identify genes likely to play a role in divergent phenotypes. We prioritized genes targeted for knockout and phenotyping by the largest phylogenetically inferred ancestral increases in gene expression across development. This approach revealed phenotypes relevant to the developmental process at a higher rate than did whole-genome or gene family knockout projects. Strikingly, a large percentage of the knockout phenotypes were concentrated in early and late perithecial development, corresponding to morphological differences between the two species: the formation of protoperithecia, and the presence of beaks and function in spore release at the late stage. Bayesian Networks predicted interactions underlying fruiting body development that differed substantially among ancestral and derived species. Gene knockouts resulted in arrested development in the species whose ortholog was upregulated in expression, but rarely also exhibited a knockout phenotype in the species whose ortholog was not upregulated. In addition, knockouts of orthologous genes in *F*. *graminearum* and *N*. *crassa* never exhibited the same phenotype, providing tangible evidence of shifts in role relating to shifts in expression. In comparing changes in expression across development between the MRCA-N and the MRCA-F, we successfully identified genes essential to several stages of sexual development. Thus we have shown that roles of genes, their expression, and their interactions have evolved along divergent evolutionary paths towards different body plans.

Our approach toward gene function discovery in the context of phenotypic evolution is more intensive than systematic approaches. Genes involved in sexual development of multicellular fungi have been identified mostly via functional analysis of gene families or genes involved in processes of interest [96–98], or by the systematic whole-genome knockout project in *N*. *crassa* [87,88]. We sorted genes with the largest difference in expression changes between the nodes of MRCA-N and MRCA-F for knockout phenotyping in *N*. *crassa* and *F*. *graminearum*, assuming that genes that diverged in expression are especially likely to be involved in morphological divergence. Compared with previous studies that systematically screened functional groups of genes, such as for transcription factors and protein kinases, for phenotypes in *N*. *crassa* and *F*. *graminearum*, our approach was more efficient in finding genes with phenotypes in sexual development. A high proportion (74%) of the knockouts in *F*. *graminearum* yielded phenotypes affecting sexual development. The characteristic of the wild type strain of *F*. *graminearum* (PH-1) to form lawns of near-synchronously developing perithecia, in the near complete absence of extraneous hyphae or conidiation, rendered phenotypic consequences of mutations particularly easy to observe.

### Roles of early and late developmental gene networks in fungal life history

Our study revealed that nearly half of the knockouts with phenotypes in sexual development in *N*. *crassa* resulted in arrest of development at the transition from protoperithecium to early perithecium, whereas the majority of knockouts with phenotypes in sexual development in *F*. *graminearum* affected numbers of protoperithecia that mature to perithecia (reducing—or in one case increasing—numbers compared to wild type). Previous studies in *N*. *crassa* also found large numbers of knockout mutants resulting in arrest of development at the transition from protoperithecium to perithecium [87,88,99–102], and previous studies in *F*. *graminearum* have revealed genes functioning in the regulation of the number of mature perithecia [96,103,104]. Previous studies have not typically found mutants conferring arrest of development at the protoperithecial stage in *F*. *graminearum* or genes functioning in regulation of the number of perithecia in *N*. *crassa*. It is possible that mutants of *F*. *graminearum* that arrest development at the protoperithecium stage would be missed in screens as a consequence of these small structures being obscured by the stroma in *F*. *graminearum*. Such mutants would be scored as completely lacking the sexual cycle. Differences in expression patterns for genes with knockout phenotypes in the initiation of protoperithecia in *N*. *crassa* were observed between the MRCA-F and the MRCA-N consistent with observed developmental differences in protoperithecia between the two lineages, implying divergent regulatory mechanisms at this stage.

The prevalence of early arrest mutants across diverse studies in *N*. *crassa* implies that the *Neurospora* species have either recently evolved or retain an ancestral regulatory apparatus that is fairly complex for entry into sexual development. For *Neurospora spp*., entering sexual development at the right time is a key life history decision. Reproductive success is contingent on remaining asexual to maximize the period of fast growth, then reliably moving into sexual development to produce robust long-lasting spores that remain dormant until the next wildfire. As organisms that thrive asexually during a brief wildfire post-burn period, then persist as durable meiotically produced ascospores [55], *Neurospora* species must grow aggressively and asexually as saprotrophs—or possibly as endophytes or pathogens [105]—then shift to the sexual cycle efficiently when nutrient resources dwindle at the end of their window of opportunity [106–108]. Each year, *F*. *graminearum* colonizes host plants and generates protoperithecia in senesced host tissues which overwinter in association with crop residues (Guenther and Trail 2005). Perithecia then arise in abundance each year during periods of optimal temperature and moisture, coinciding with host development to allow for maximum infection. Thus the sexual cycle of each species is initiated in coordination with diminishing nutrients (senesced host tissues in *F*. *graminearum* versus post-fire woody plant exudates in *N*. *crassa*) and environmental signals. Expression patterns of genes with phenotypes in early perithecium development appeared similar among the *Neurospora* and *Fusarium* species, consistent with the similarity of early morphologies of perithecial development, although the greater diversity of early perithecial phenotypes among *F*. *graminearum* genes implies that its genes operate in a more decentralized regulatory context.

Beak development and spore release phenotypes manifested in *N*. *crassa* and *F*. *graminearum* respectively at Stage 5, yet these phenotypes originate in different tissues, apparently by the operation of non-homologous genes, presenting a dichotomy in phenotypes observed in the two species. The Bayesian Networks generated from genes whose knockouts interrupt late perithecial development in both *Neurospora* and *Fusarium* put forth a hypothesis that gene function and pathway interactions have evolved. In particular, the regulation of spore release in MRCA-F could have diverged from that in MRCA-N, segregating into two distinct components in *Fusarium*, but being maintained as an integrated network in MRCA-N (**Fig 6**). The present study identified four genes that affect spore release in *F*. *graminearum*: *rel-1*, *rel-2*, *rel-3*, and *rel-4*. The hypothesized Bayesian network parallels experimental evidence: *mid1* and *cch1* are inferred to interact directly in the MRCA-F and in the MRCA-NF, whereas they do not manifest direct interactions in the Bayesian network inferred for the MRCA-N. Two genes, *mid1* and *cch1*, involved in calcium regulation, have been previously identified in *F*. *graminearum* as being essential for ascospore release [109], and knockouts of these genes in *F*. *graminearum* suggest that Mid1 functions upstream of Cch1 [77–81]. In contrast, the *N*. *crassa mid1* knockout was wild-type for spore release [82,109]. This discrepancy in function inspires consideration of the relation between divergence of the Bayesian networks and divergence of function in sexual development. Bayesian Networks based solely on wild type gene expression measurements are hypothetical until verified by additional gene-specific functional studies. Their combination with knockout phenotypes can help us build a more rigorous understanding of the relationships among genes important to a certain morphological process and how those relationships evolved.

In the ascomycetes, the fruiting body wall is thought to be formed from maternal tissue [90,105,110,111]; thus, regulation of wall development (including beak development) might proceed independently of the development of the perithecial contents, such as ascospores. Accordingly, there are no previous reports of any developmental linkage between beak development and asci/ascospores production and release. However, in six of our knockout mutants that affected spore development, all but one also affected beak development, supporting an argument that beak and spore development are tightly coordinated in *N*. *crassa*. Of eight other genes examined in recent studies whose knockouts have been attributed absence or reduced beak phenotypes [87,109,112], six also produce no ascospores [including transcription factors bek-1 and bek-2; 113], and two others produce additional knockout phenotypes observable throughout perithecial development. The developmental linkage between ascospore-deficiency and beak phenotype is asymmetrical: in 151 other knockout strains with ascospore knockout phenotypes, not a single beak mutant phenotype was reported [109,112]. A developmental linkage between beak development, ascospore development, and potentially earlier stages of perithecial development argues for a highly integrative gene network in which key genes play developmental roles across time and in diverse developmental structures. Such an integrative network would demand coevolutionary changes in multiple genes within the network to produce useful novel phenotypes, a demand that is consistent with the degree of divergence in developmental function of genes between *N*. *crassa* and *F*. *graminearum*. Although genes essential to beak development in *N*. *crassa* are present in *F*. *graminearum*, their hypothetical network interactions are more distant. Indeed, *F*. *graminearum* knockouts of five of the genes essential to beak development in *N*. *crassa* exhibited phenotypes at diverse stages of sexual development. Only the knockout of *stc1* yielded a common stage of arrest in *N*. *crassa* [99] and in *F*. *graminearum*, although the expression patterns of the orthologous genes and phenotypes of orthologous knockouts in the latter were different. All other genes in the network exhibited a wild type knockout phenotype in *F*. *graminearum*, indicating that their roles in these processes are either ancestral functions in the MRCA-NF that have been selectively retained in *N*. *crassa* or they are novel to *N*. *crassa* and perhaps its close relatives.

Both *Neurospora* and *Fusarium* species release spores by forcible discharge to achieve long distance dispersal of sexual propagules, however the context of that release and the role of release in the life cycle of the species differ. For *F*. *graminearum*, the initiation of the perithecial development from protoperithecia commits the fungus to a continuous developmental trajectory through spore release [36]. The requirement of light for initiation [114,115] necessitates perithecium formation on above-ground plant tissues and crop residues positioned on the soil surface. Perithecia of *N*. *crassa* are initiated underneath a layer of plant tissue, and the beak is used to breach the surface and eject spores into the free air for dispersal [116]. The beaks are phototrophic, and they must be fully mature for the spores to fire. Thus, regulation of spore release coincides with the decision to engage in sexual development in *F*. *graminearum*, and is regulated later in sexual development for *N*. *crassa*, permitting each to tune its regulation to optimize niche adaptation.

### Divergence in gene expression and function in development

Previous research has demonstrated that morphologically homologous structures can retain a remarkable degree of phenotypic conservation in multiple lineages despite dramatic divergence of the underlying pathways and gene interactions [i.e. “developmental systems drift”: 117,118–121]. Among the *N*. *crassa* orthologs of the 18 genes exhibiting knockout phenotypes in sexual development in *F*. *graminearum*, a majority (14 of 18), exhibited wild type knockout phenotypes. This divergence between species of the map between a phenotype close to the genotype (gene expression) and the complex macroscopic phenotype of fungal fruiting body development indicates a striking change in the role of these genes in one species over the other, supporting the hypothesis that these genes have diverged and become specialized in *F*. *graminearum* related to sexual development. The *F*. *graminearum* orthologs have adopted nonredundant essential roles in development, whereas the *N*. *crassa* orthologs either no longer have roles in development or now have nonessential roles. In a minority of cases (2 of 18), the *N*. *crassa* knockout phenotype, although different in kind, occurred at a similar stage of development to that of the orthologous knockout phenotype in *F*. *graminearum*.

In two other cases, knockouts of orthologous genes in *N*. *crassa* and *F*. *graminearum* exhibited phenotypes that were different in stage as well as kind. In the first case, knockouts of *pna-2* in *N*. *crassa* were arrested in Stage 3, forming no beak or asci, whereas knockouts of the ortholog in *F*. *graminearum* were arrested at Stage 4, forming asci with spores that never matured. The manifestation of these knockout phenotypes at similar stages in the two species suggests that the orthologs have retained some similarity of function since their divergence approximately 220 million years of evolution [38; Fig 1]. The slightly earlier arrest of Δ*pna-2* in *N*. *crassa* is evidence of the high functional importance of the up-regulation of the gene starting just before Stage 3—an expression pattern shared with many meiosis and asci/ascospore development genes [99]. While upregulation of *pna-2*, as well as *stc1* and *pna-1*, persisted through Stage 5 in *N*. *crassa*, the down-regulation following Stage 3 of the *F*. *graminearum* orthologs to these three genes is notably divergent. In a second case, expression patterns over development of *lpe-1* in *Fusarium* and its ortholog in *Neurospora* were similar, with expression in *Fusarium* species peaking about 20-fold higher. Nevertheless, phenotypes of the knockouts were different in stage as well as kind: the knockout of *lpe-1* in *F*. *graminearum* resulted in reduced numbers of perithecia and very slow maturation, whereas the knockout of the ortholog in *N*. *crassa* exhibited abnormal ostiole morphology and no ascospore production. In the evolution of this pair of orthologs, the genes in the two different fungi have markedly diverged in their impact on phenotype within the same developmental process.

Our results suggest that products of development such as the *Neurospora* beak that result from complex life-history decisions in response to ecological factors exhibit greater variability than do products of invariant, non-plastic developmental programs. One potential source of this variability is a high regulatory density to the trait—that is, the large number of genes that likely contribute to complex ent. Whether or not the evolution of new phenotypes requires the modification of suites of interacting genes coordinated by developmental processes [39], this bias in variability, correlated with regulatory density that would arise as a consequence of environmental modulation, could contribute substantially to phenotypic diversification, speedily constructing novel, extensive, functionally integrated phenotypes. Identifying these differences in variability and mapping variability to its source is key to understanding why developmental evolution of some traits is very hard, and developmental evolution of other traits is comparatively easy.

### Conclusions

In this study we have shed light on the evolution of the “black box” between genotype and phenotype [30] in fungal sexual development. Beyond identifying individual genes and their phenotypes, approaching comparative studies with functional assays guided by evolutionary analysis helps to address the challenge of dissecting emergent properties. Specifically, while identifying the components that play a role in the development of morphologies of interest, this inherently comparative approach also provides insight into larger-scale dynamics of gene interaction and network output. In the case of fungal fruiting body development within the Sordariomycetes, we have shown that few developmental genes whose expression evolves retain common phenotypes or even phenotypes corresponding to a common stage of development, yet numerous genes do retain some function within the the larger fruiting body developmental process. Correspondingly, we have shown that phenotypes such as the beak in *Neurospora* or spore release in *Fusarium* can dramatically shift their genetic architecture while still being based largely on genes involved in the wider developmental process of fruiting body development, providing potential for the evolution of novel phenotypes. Gene expression data provide a means for systems-level integration of information, and an evolutionary framework provides a meaningful integration of analyses spanning genetic, developmental, phenotypic, biodiversity, and phylogenetic data, with evolutionary thinking at its core. We have revealed key genes, their relevance to sexual development, and their lability in the network regulating fungal sexual reproduction using an efficient systematic approach empowered by comparison and contrast of gene expression programs across multiple taxa. Mapping differential variational properties associated with developmental processes represents a critical component to understanding how and why developmental evolution occurs the way it does. The concentration of genes identified as having a role in early and late perithecium development may support an hourglass model for development, which has recently been suggested to operate in mushroom-forming fungi [122]. Our study demonstrates a new and powerful approach to identifying genes that function within a developmental process that could be applied to future research integrating evolutionary study of the development of multiple taxa in response to relevant environmental cues.

## Materials and methods

### Strains and culture conditions

To generate transcriptomes (**Fig 2**), strains of *N*. *crassa* complementary mating types *mat a* and *mat A* (FGSC4200, FGSC2489 respectively) and knockout strains from the *N*. *crassa* knockout project [87,109,112] were obtained from the Fungal Genetic Stock Center [fgsc.net; 123]. *N*. *crassa* is self-incompatible, meaning that strains of the opposite mating type are essential for fruiting body maturation. *F*. *graminearum* is self-compatible, but can outcross [112]; strain PH-1 (FGSC9075, NRRL31084) has been previously described [51]. *F*. *graminearum* strain PH-1-55 is a *nit-3* mutant of the PH-1 strain, unable to use nitrate as the sole nitrogen source (isolated by Talma Katan and provided by H. C. Kistler), which we have previously used to generate recombinant progeny [109]. Stocks of *N*. *crassa* were stored on slants at –20 C and the knockout mutants were retrieved from stocks stored at –80 C. *F*. *graminearum* strains were stored as conidial stocks (10^6^ conidia / ml) or as blocks of Carrot Agar [124] colonized with mycelia in 35% glycerol stored at –80 C. Phenotypes in sexual development were assayed on Synthetic Crossing Medium [125] and CA for *N*. *crassa* and on CA for *F*. *graminearum*. In *N*. *crassa*, the sexual stage was initiated by crossing the mating type strains (*mat A* and *mat a*) on SCM, and protoperithecia and perithecia which developed along the crossing zone were examined. In *F*. *graminearum*, sexual development was induced by spreading aqueous 2.5% Tween 60 across the surface of the culture with a sterile glass rod as previously described [83]. The term protoperithecium indicates the unfertilized fruiting body initial produced by the female parent. We use the term protoperithecium for the very young perithecium initials in *F*. *graminearum* even though it is self-compatible, and fertilization is not essential for fruiting body development.

### Identification of orthologous genes and comparative gene expression

To identify orthologs for comparison of gene expression (**Fig 2**), protein and nucleotide sequences for the five *Neurospora* and *Fusarium* genomes were downloaded from the Broad Institute genome database (http://www.broadinstitute.org). Predicted protein sequences were used to identify single copy orthologs (cluster) with ReMark [126]. ReMark was executed specifying the BLOSUM62 amino acid transition matrix [127] and an inflation factor of 1.6—selected so as to make conservative ortholog calls. Additional orthologs among the five genomes were identified using BranchClust [86] based on nucleotide data (**Fig 2**). The sensitivity of our detection of ortholog sets was validated by comparison to those reported in the FungiDB [128]: all single copy ortholog sets in FungiDB were also identified by our analysis. To estimate the accuracy of ortholog calls, 50 predicted ortholog sets, including genes belonging to large gene families, were further verified with manually conducted phylogenetic analyses based on results from exhaustive BLAST searches. We have previously conducted whole genome expression analysis across perithecium development in *F*. *graminearum* and *F*. *verticillioides*, and in *N*. *crassa*, *N*. *discreta*, and *N*. *tetrasperma* [48,49]. Data for gene expression levels have been deposited in NCBI (https://www.ncbi.nlm.nih.gov/geo) for the *Neurospora* species (GSE60255, 60256 and 60257), and the *Fusarium* species (GSE61865). Here we report results on those predicted orthologs for which expression was detected across all sampled stages in all five species. In this research, we did not further consider the orthologous genes that were multi-copy in some species. While there are very interesting questions to ask regarding genes with multiple copies, their evolution and the evolution of their level of gene expression is affected by additional considerations [129–134], and it is not possible to use these multi-copy genes to address our goal in this manuscript.

### Continuous ancestral state inference

To infer ancestral states for the continuous character of gene expression (**Fig 2**), a phylogeny of the five species (**Fig 1**) was inferred from the protein sequence alignment of the largest and the second largest subunit of DNA-directed RNA polymerases, RPB1 and RPB2, analyzed with PAUP 4.0* [135]. Using the maximum likelihood topology and branch lengths generated, an ultrametric tree was produced using r8s, estimating divergence times with a penalized likelihood model using the TN algorithm [136]. Transcriptomic analyses of gene expression across five or more developmental stages for each genus cultured on a common CA medium have been previously published [48,99]. We maintained a common medium across species, which was necessary for inference of evolved differences, as external environmental conditions significantly influence gene expression if not morphological development [137].

The systemic, gene-interaction-based developmental output of sexual phenotype depends on both cis- and trans- regulation, but is more directly related to trans-regulation. Whereas cis-regulatory gene expression/functional evolution might be directly related in magnitude to gene tree topology and branch lengths as opposed to species-tree topology and branch lengths, trans-regulation should not be. Therefore, for all analyses, all of our analyses are based on the well-accepted species phylogeny inferred from informative genes [138,139]. These species are sufficiently diverged from each other that there are unlikely to be significant discrepancies between gene trees and the species tree, with the exception of the special case of the mating type genes and mating pheromone genes within the most closely-related clade of Neurospora species [*N*. *crassa*, *N*. *discreta*, and *N*. *tetrasperma*; 140].

Relative gene expression levels across all developmental stages for orthologs in each of the five species were estimated using LOX [Level Of eXpression; 141]. From the relative gene expression across stages for all single copy orthologs, we calculated the fold-change in expression between each adjacent pair of stages, which we preferred to use over “absolute” level of gene expression for several reasons: 1) we have no way to calibrate “absolute” gene expression levels between species—we would have to arbitrarily assign equality of expression at an arbitrary stage of development, a decision that would affect our results; 2) Measures of “absolute” gene expression level—across *all* technologies—have been notoriously poor and poorly correlated across technologies, whereas relative gene expression levels have been much more reliable both within a technology and between technologies applied to the same sample [142–144], largely because relative expression from stage to stage is internally controlled within the experiment; and 3) there is no reason to view relative changes as less relevant, less fundamental, or less important than the gene expression “levels” themselves—in fact, there is reason to believe that the stage to stage changes may be more informative about biology than absolute levels would be, because gene expression is typically a highly dynamic response to environmental and developmental state [145–147].

The fold-change between stages and the molecular evolutionary tree were supplied as input files to the Continuous Ancestral Character Estimation [CACE; 148, 149] tool in the Discovery Environment Application list in iPlant [150–152], which provided ancestral changes in expression across adjacent stages at all internal nodes for every ortholog set. CACE applies a Brownian motion model, parameterized with a rate for each gene, calculating the ancestral expression values by maximum likelihood under the model assumption that the expected squared differences between any two species is the rate multiplied by the time since the species last shared a common ancestor [148]. There is some degree of uncertainty associated with the measurements of gene expression, as well as high uncertainty (on top of that measurement error) with regard to the estimate of ancestral state. It is possible to integrate over these uncertainties [c.f. 145]. However, such integration over uncertainty would not affect the estimate. Performance of this computationally intensive resampling would leave the primary objective of this analysis pipeline (our ranked list of the genes estimated to experience the greatest changes in stage to stage gene expression) unchanged, because the mean of serially sampled uncertainty distributions is unchanged by changes to the higher moments of the distributions, and only higher moments of the distributions would be affected by the serial resampling that could reflect these uncertainties.

We chose to use CACE on individual genes so as to account for phylogenetic dependence associated with phylogenetic structure and divergence (Felsenstein 1985; Martins 1996; Felsenstein 1988) in the estimation of the continuous character of ancestral expression of each gene (Schluter et al. 1997). This model-based approach incorporating phylogenetic divergence (branch lengths) has been demonstrated to fare much better for the purpose of comparison of traits across species than does assuming complete independence (Felsenstein 1985; Martins 1996; Felsenstein 1988) or assuming complete dependence and averaging quantitative characters across taxa within clades. By estimating ancestral expression at the individual gene level, we are able to independently identify genes whose expression during development changed markedly along the phylogeny. We can then knock them out, ascertain their function in development by phenotyping the knockout, and use that knowledge to assign the genes to common developmental modules. Our approach stands in contrast to the common practice of assigning developmental modules based on correlation of gene expression, whether performed on data from single species (Eisen et al. 1998; Hughes et al. 2000) or on data from multiple species informed by a phylogeny (Thompson et al. 2013; Roy et al. 2013; Knaack et al. 2016; Thompson et al. 2015). Whereas we are using assayed developmental function to define the gene content of our modules, these approaches use correlation of gene expression pattern to define the gene content of modules.

For display of ancestral expression levels, inferred ancestral changes in expression across adjacent stages were then transformed back into relative expression measures across stages, with the lowest expression across stages set to one.

### Candidate genes for functional investigation

To select genes that pertained to developmental differences observed between *Fusarium* and *Neurospora* species, estimated ancestral expression was contrasted between MRCA-F (**Fig 1**) and MRCA-N (**Fig 1**), sorting all genes by their largest evolved difference in change of expression between stages (*t*), calculated as (*X*_*t+1*_*-X*_*t*_) / *min* [*X*_*t*_, *X*_*t+1*_], where *X* is the expression level. When that difference represented an increase in expression in the MRCA-F relative to MRCA-N, the genes were correspondingly ranked for a priority list of knockout candidates in *F*. *graminearum* (**Fig 2**). When that difference represented an increase in expression in MRCA-N relative to MRCA-F, the genes were correspondingly ranked for a priority list for knockout mutants in *N*. *crassa*. In *N*. *crassa*, the knockout strains that were available from a gene deletion project [87,109,112] were investigated from the top 100 genes and reported in this study. In *N*. *crassa*, where we had scope for performance of additional knockout phenotyping, we also independently prioritized genes within each stage by difference in estimated expression level between the MRCA-N and the MRCA-F. We examined 45 genes from this additional priority list, even though these two strategies were prone to identify highly overlapping gene sets. Also, using a smaller set of high-confidence orthologs that were identified by a phylogenetic approach as in Lehr et al. [48], we selected 70 genes in *N*. *crassa* and 23 genes in *F*. *graminearum* for knockout phenotype investigation, and prioritized the largest within-stage differences between the estimated expression levels in the MRCA-N and in the MRCA-F, across all stages.

### Nucleic acid manipulation and genetic transformation

Knockouts were performed in *F*. *graminearum* or examined in *N*. *crassa* with strains obtained from the *Neurospora crassa* knockout project [87,109,112]. To generate gene-replacement strains in *F*. *graminearum*, we used a split marker approach in which the left and right flanking regions of the target gene were amplified and fusion PCR was used to merge each with a portion of the genetic marker gene (*hph* encoding hygromycin phosphotransferase from *E*. *coli* under control of the *trpC* promoter and terminator from *Aspergillus nidulans* [153]). Fusion PCR was used to generate constructs with target gene-specific primers (**S8 Table**) and performed as described previously [154]. Gene models and genomic sequence for the primer design were obtained from the Munich Information Center for Protein Sequences (MIPS) *Fusarium graminearum* Genome Database [version 3.2; 155]. The two fusion fragments were then pooled and introduced into *F*. *graminearum* by polyethylene glycol mediated transformation of protoplasts [156] as modified by Cavinder and Trail [157]. Following transformation, hygromycin resistant putative transformants were examined for loss of the appropriate gene by PCR check. Two checks were used, one in which the presence of the hygromycin gene was confirmed in the knockout, through use of the hygromycin primers (“Univ Hyg F” and “Univ Hyg R”; **S8 Table**), and a second check, in which primers outside the area of gene replacement were chosen to amplify the entire region (**S8 Table**), documenting the gene replacement by a shift in size (**S4 Fig**). A minimum of two PCR-confirmed knockout mutants were obtained and examined for phenotypic differences from wild-type. Where more than two were available, all were examined.

Perithecium formation in the knockouts was examined by stereomicroscopy for shape, size, number formed, cirrhus formation (ascospores oozing *en masse* from the perithecium), and for changes in beak development (in *N*. *crassa* only). Squash mounts of perithecia in water were examined using a compound microscope for the presence and maturity of asci, ascospore shape, number per ascus, and presence of paraphyses. Ascospore discharge was evaluated by checking the lid of the Petri dish in mature cultures for presence of ascospores. Beak development was quantified for mutants showing beak phenotypes in *N*. *crassa*, where perithecial maturation and beak development were closely monitored. Perithecia were sampled and measured when no apparent size changed in 10 days after crossing for all isolates. Twenty random perithecia were sampled across two plates for each isolates. The length of the beak, the width of the beak base, the length of the perithecium (including beak), and the width of the perithecium were recorded under a dissecting microscope (10× magnitude).

Knockout mutants for four genes from *F*. *graminearum* (*rel-2*, *asy-1*, *stc1*, and *pdv-2*) were individually crossed with strain PH-1-55. Cirrhi from single perithecia along the intersection of the two strains (as *F*. *graminearum* is primarily self-compatible, but can outcross) were collected to analyze segregation of the *nit-3* from hygromycin resistance and the knockout phenotype. Crosses were accomplished and progeny selected as previously described [87,109,112], using chlorate containing medium to detect nitrate reductase (*nit-3*) mutants. The presence of *hph1* was confirmed by PCR as described above using gene-specific primers (**S8 Table**). For each of the four genes, at least 30 progeny (15 NIT^+^ and 15 NIT^−^) were examined. Cosegregation tests were used to confirm that the observed phenotypes were caused by knockout of the targeted genes (**S4 Table**). All hygromycin resistant cultures arising from these ascospores exhibited the phenotype of the knockout parent, indicating that the observed phenotype was linked to hygromycin resistance as a result of deletion of the target gene [87,88,99–102]. For knockout strains that produced phenotypes that arrested at protoperithecia or before ascospore development, conidia were isolated from the knockout strains and used to fertilize protoperithecia from the wild type strains. To assess association of the knockout phenotype with Hyg^r^ (*hph1* being used as the selectable marker at the location of the deletion mutation), Hyg^r^ cultures were isolated from 17–28 ascospores from each cross.

Frequencies of knockout phenotypes in *F*. *graminearum* at each stage were compared to frequencies of knockout phenotypes at each stage in two large knockout projects in *F*. *graminearum* (**Fig 4**)—kinases [97] and transcription factors [96]. Statistical significance was evaluated by a two-tailed Fisher Exact Test applying a threshold *α* = 0.05.

### Bayesian network prediction for early and late perithecial development

Bayesian networks were generated to reveal gene networks or modules involved in the evolutionary changes in morphology [30] along the phylogeny of *Neurospora spp*. and *Fusarium spp*. Biological networks were modeled using the Bayesian Network Web Server [158] supplied with expression data for each sampled species and ancestral node of genes whose knockouts exhibited phenotypes in early perithecial development of *N*. *crassa* and *F*. *graminearum* (**S9 Table**). Bayesian networks were also generated for genes showing phenotypes in late perithecial development, including beak formation in *N*. *crassa* and cirrhus production in *F*. *graminearum*. Input files contained fold changes between adjacent sample points across the experiment (across eight developmental stages for *Neurospora* species, six developmental stages for *Fusarium* species, and five developmental stages for ancestral nodes). Global structure learning settings were retained at default settings. The models depicted are the 50% majority consensuses of 100 models (selection threshold set to 0.5, the 100 highest-scoring networks were averaged), without imposing any structural constraints. Note that evolution of the Bayesian networks along the phylogeny reflects differences in regulatory interactions between genes. Bayesian Networks inferred would be unchanged if there were differences in expression in individual genes that—because of other un-depicted regulatory alterations—alter individual gene expression but not the gene interactions among those genes depicted.

## Supporting information

S1 Fig Phenotypes of knockout strains in *Fusarium graminearum*. (PDF)

S2 Fig Phenotypes of knockout strains in *Neurospora crassa*. (PDF)

S3 Fig Beak measurements for *Neurospora crassa* wild type, and knockout mutants identified in this study as exhibiting short beaks. (PDF)

S4 Fig PCR verification of knockout strains in *Fusarium graminearum*. (PDF)

S1 Table Clusters of orthologous genes for the five species of Neurospora and Fusarium. (XLSX)

S2 Table Estimated gene expression for the most recent common ancestors. (XLSX)

S3 Table Ranked genes based on the difference in expression between the Fusarium MRCA and Neurospora MRCA. (XLSX)

S4 Table Segregation analysis of knockouts of four genes in *Fusarium graminearum*. (XLSX)

S5 Table Ranked genes based on the difference in expression between the Neurospora MRCA and Fusarium MRCA. (XLSX)

S6 Table Identification of orthologs in *Saccharomyces cerevisiae* of genes knocked out in *Neurospora crassa*. (XLSX)

S7 Table Predicted networks of genes with phenotypes in perithecial development in *Neurospora crassa* and *Fusarium graminearum*. (DOCX)

S8 Table Primers used for gene knockouts in *Fusarium graminearum*. (XLSX)

S9 Table Bayesian network input files for the F-MRCA, N-MRCA, and FN-MRCA, the most recent common ancestor nodes for 2 Fusarium, 3 Neurospora, and all 5 Fusarium and Neurospora species. (XLSX)
