# Supplementary material for: The ancestral levels of transcription and the evolution of sexual phenotypes in filamentous fungi
Source: PLoS Genet. 2017 Jul 13;13(7):e1006867. doi: 10.1371/journal.pgen.1006867 (PMC5509106; doi:10.1371/journal.pgen.1006867)

**S1 Fig. Phenotypes of knockout strains in *Fusarium graminearum***

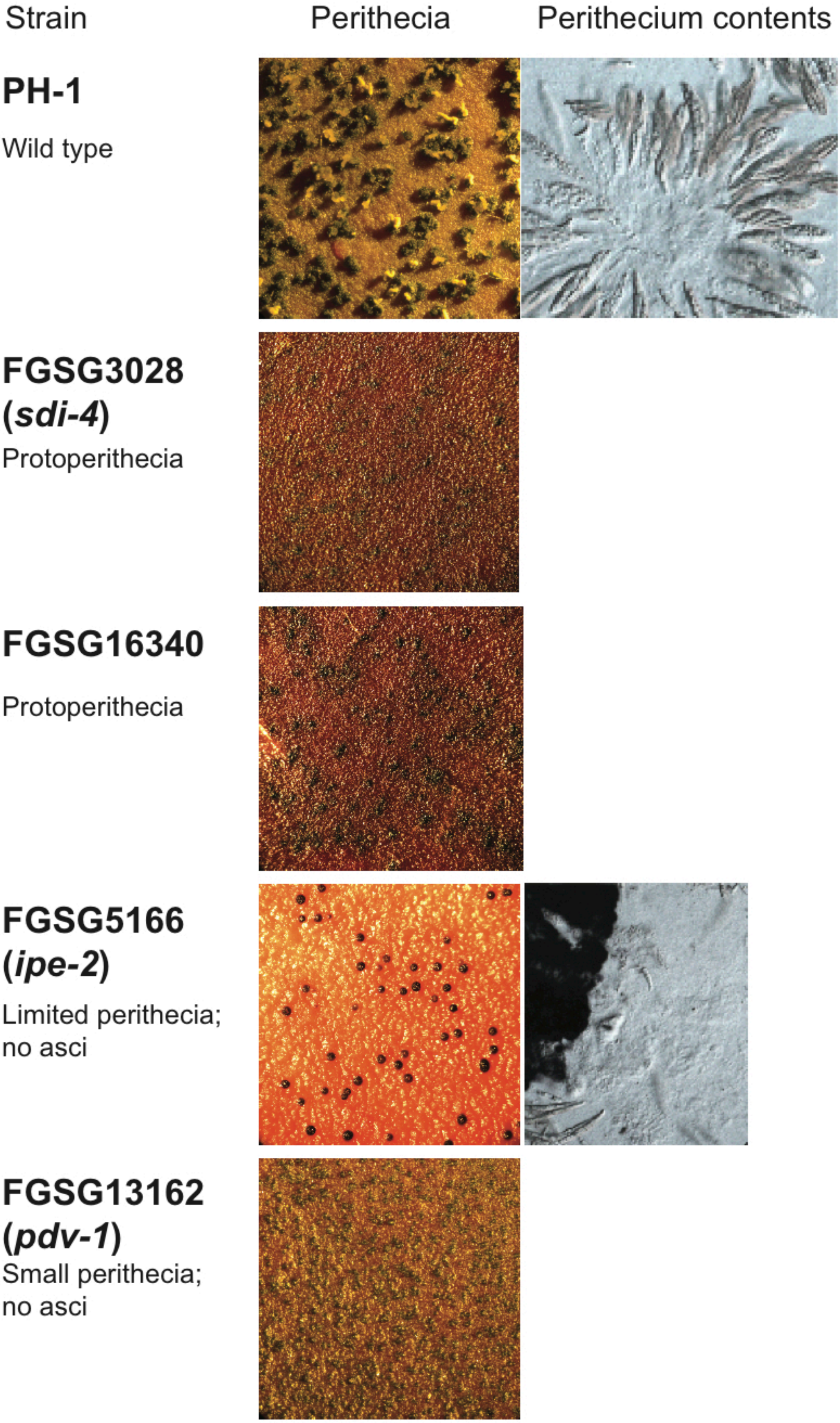

**FGSG2102 (*asy-1*)**
Asynchrononus development; mature normally

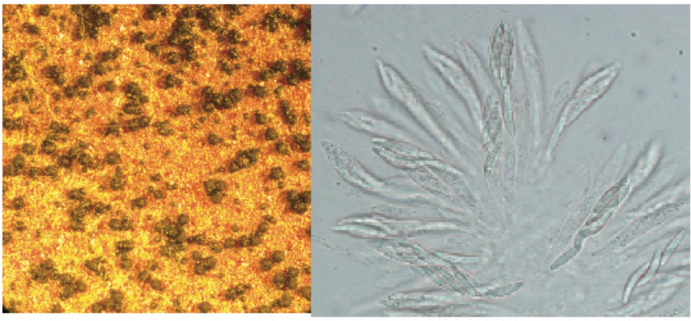

**FGSG0565 (*div-12*)**
Limited perithecia; delayed development

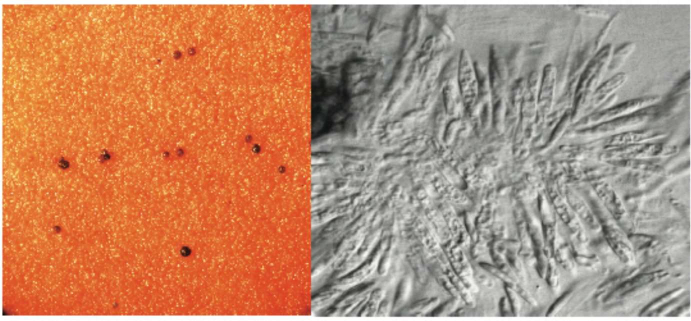

**FGSG6651 (*vad-1*)**
Limited numbers of perithecia; delayed development,minimal cirrhi production

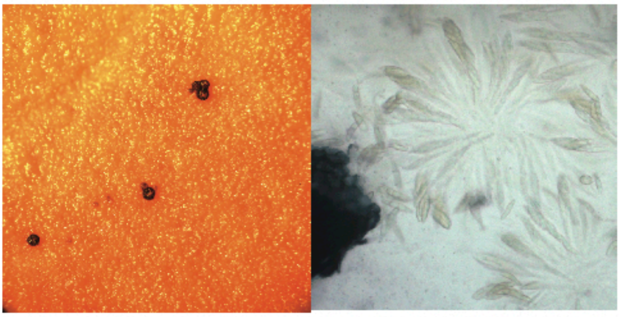

**FGSG7478 (*ipe-1*)**
Limited perithecia; delayed development

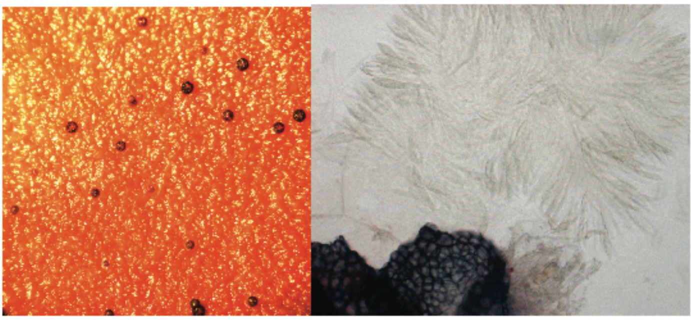

**FGSG8695 (*pls-1*)**
Increased number of perithecia

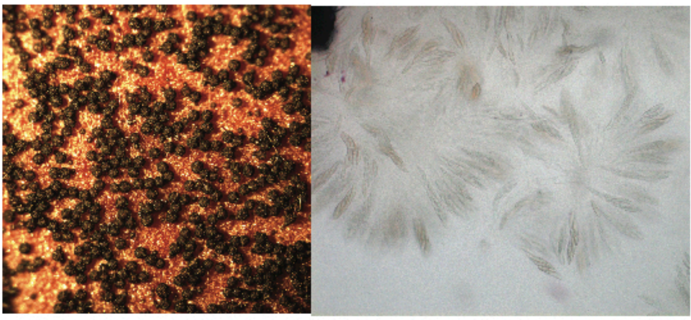

**FGSG4997 (*pna-2*)**
Some asci are underdeveloped; no cirrhi; less firing.

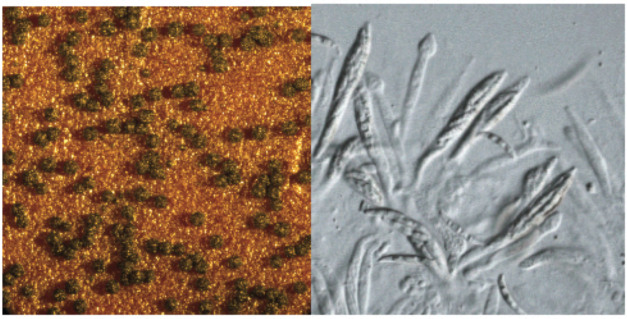

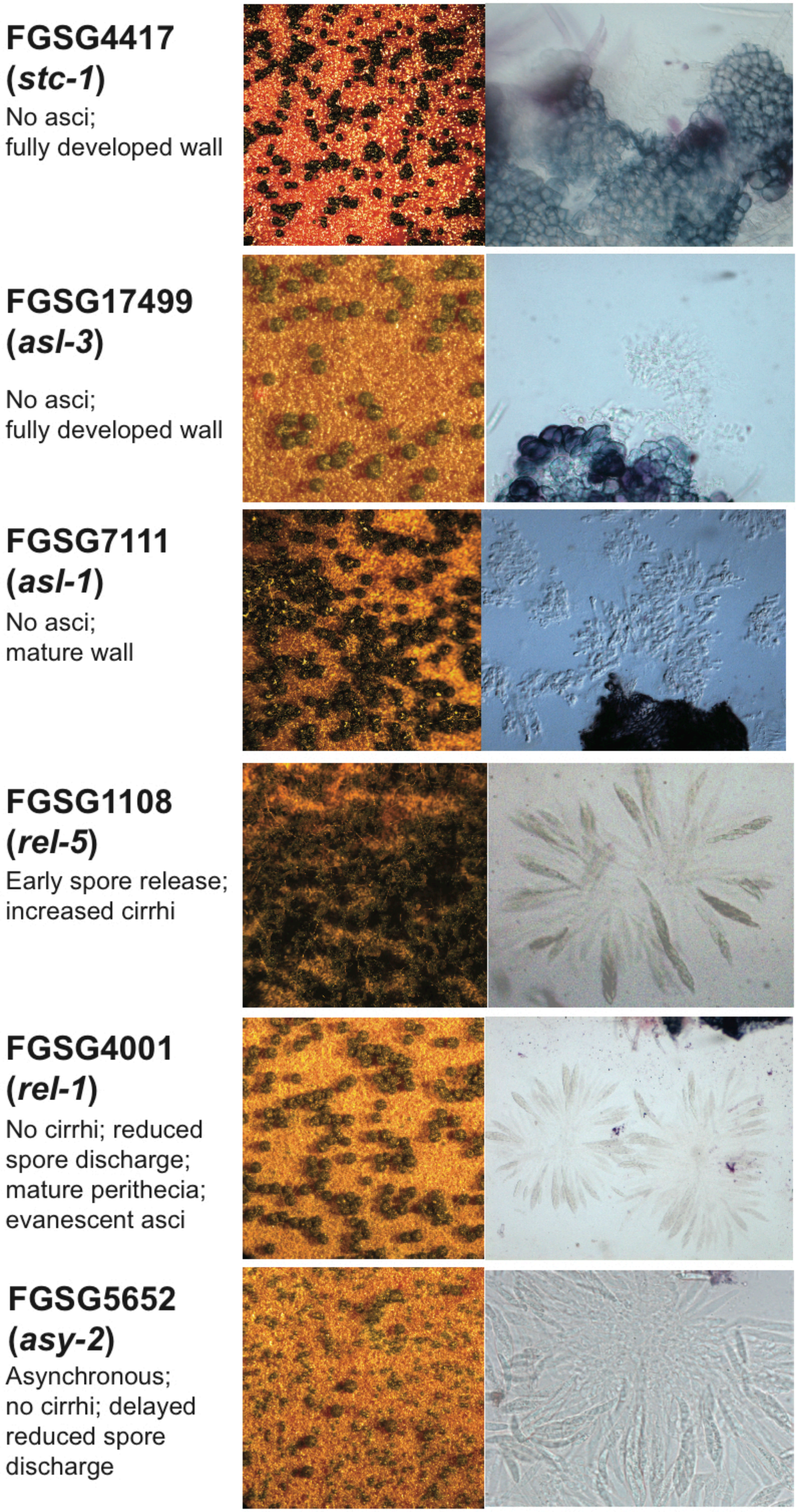
FGSG4417
(stc-1)
No asci;
fully developed wall
FGSG17499
(asl-3)
No asci;
fully developed wall
FGSG7111
(asl-1)
No asci;
mature wall
FGSG1108
(rel-5)
Early spore release;
increased cirrhi
FGSG4001
(rel-1)
No cirrhi; reduced
spore discharge;
mature perithecia;
evanescent asci
FGSG5652
(asy-2)
Asynchronous;
no cirrhi; delayed
reduced spore
discharge

**FGSG10094**

No cirrhi;
reduced firing

**FGSG17494**
**(*rel-4*)**

Reduced firing;
no cirrhi

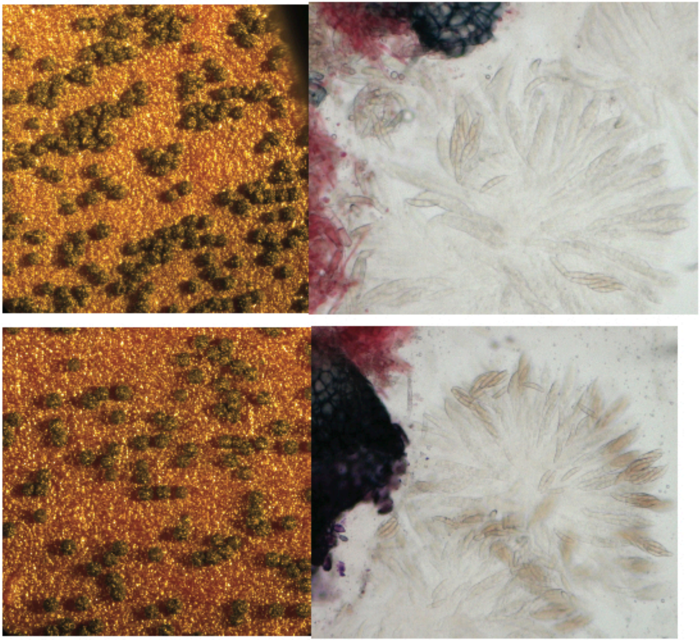

Supplement: S1 Fig — (PDF) [file pgen.1006867.s001.pdf]
