# Supplementary material for: The ancestral levels of transcription and the evolution of sexual phenotypes in filamentous fungi
Source: PLoS Genet. 2017 Jul 13;13(7):e1006867. doi: 10.1371/journal.pgen.1006867 (PMC5509106; doi:10.1371/journal.pgen.1006867)
Supplement: S2 Fig — (PDF) [file pgen.1006867.s002.pdf]

S2 Fig. Phenotypes of knockout strains in *Neurospora crassa*

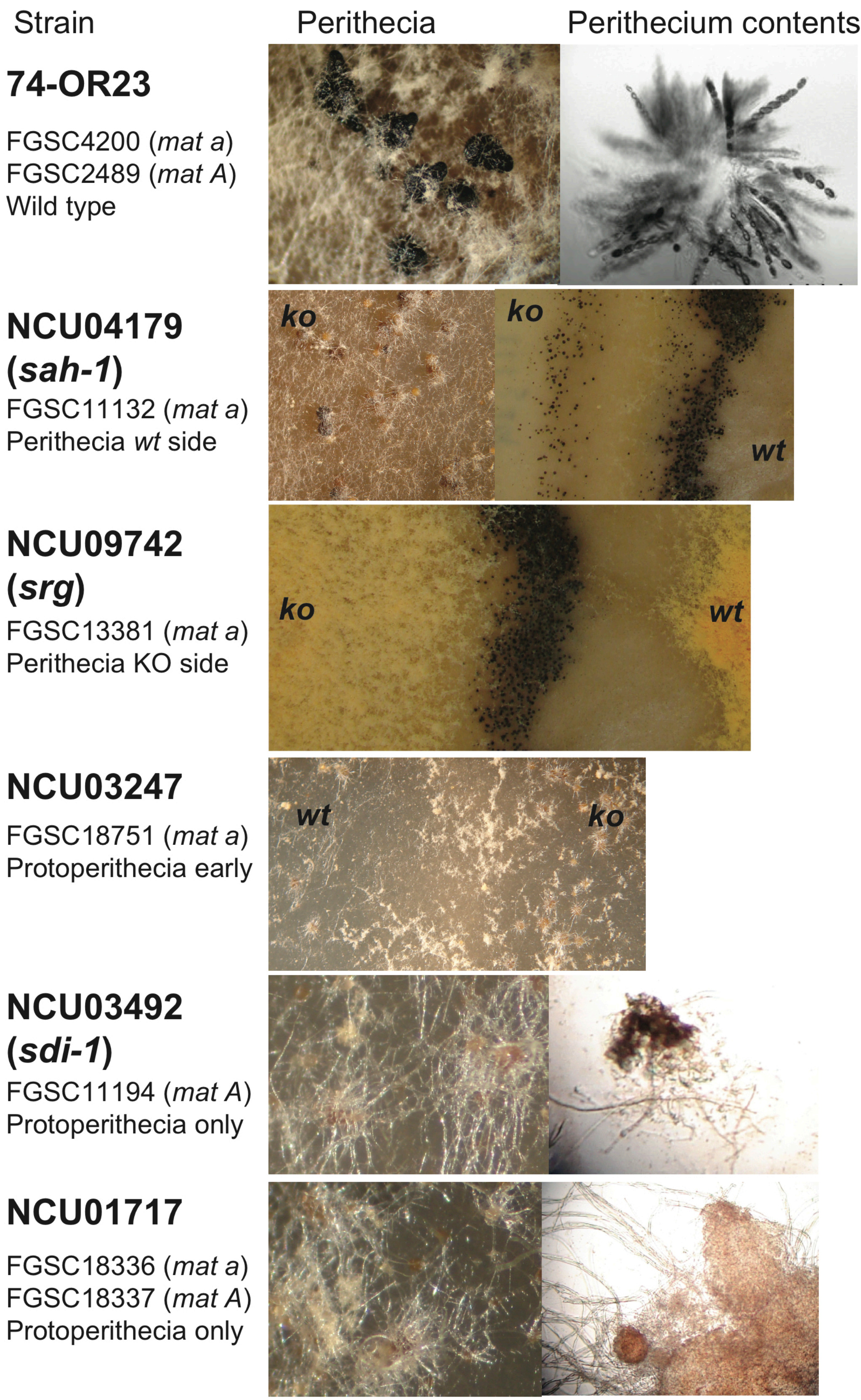

NCU02089

FGSC19087 (*mat a*)
FGSC19088 (*mat A*)
Protoperithecia only

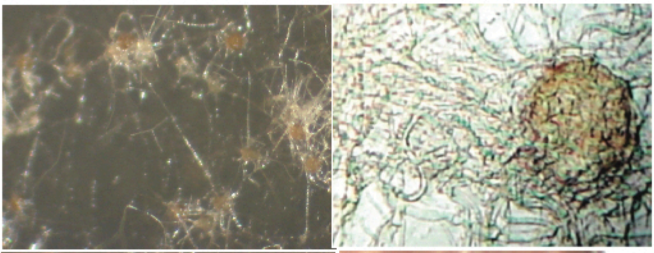

NCU03121

FGSC14338 (*mat a*)
FGSC14339 (*mat A*)
Protoperithecia only

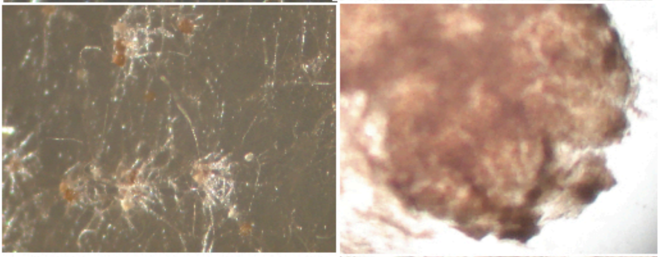

NCU04713

FGSC19138 (*mat a*)
Protoperithecia only

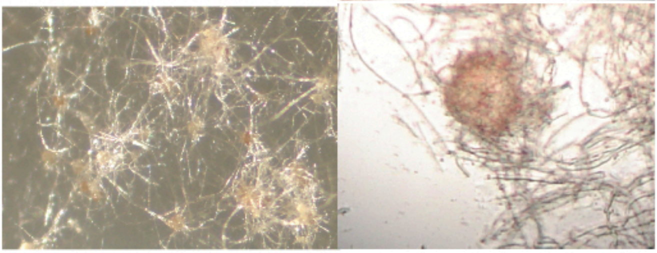

NCU04744 (*sdi-2*)

FGSC19139 (*mat a*)
Protoperithecia only

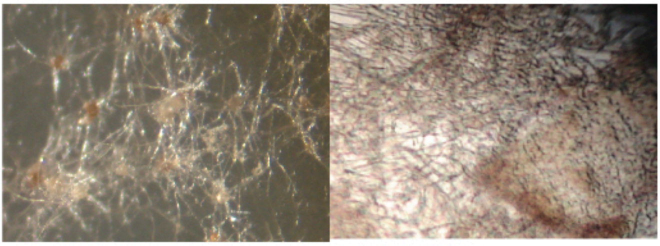

NCU05882 (*sdi-3*)

FGSC19950 (*mat a*)
Protoperithecia only

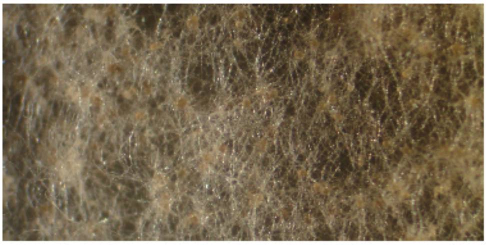

NCU07441

FGSC15502 (*mat a*)
FGSC15503 (*mat A*)
Protoperithecia only

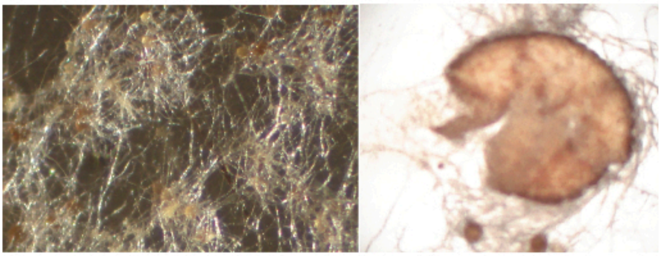

NCU07449

FGSC13456 (*mat a*)
FGSC13457 (*mat A*)
Protoperithecia only

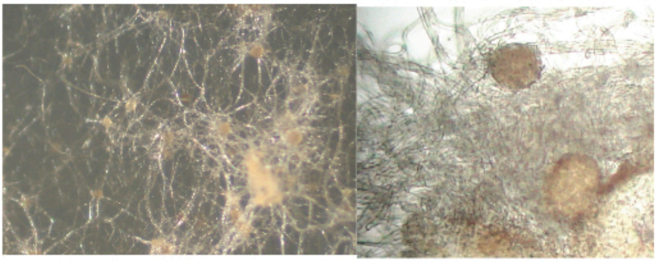

NCU02879 (*sbk-1*)
FGSC12600 (*mat a*)
FGSC12601 (*mat A*)
Short, small beak

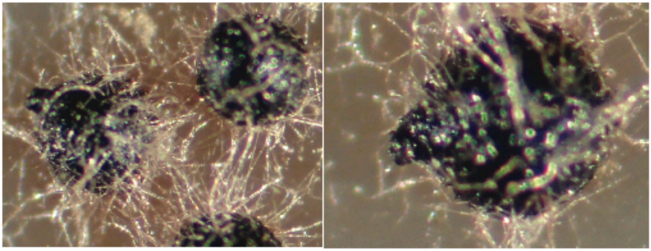

NCU05858
FGSC11242 (*mat A*)
Short, small beak

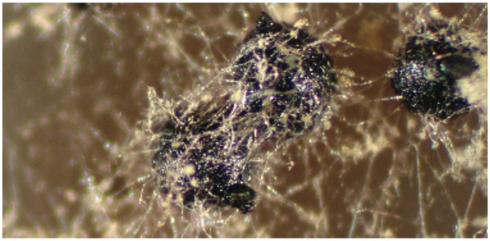

NCU07621 (*tzn-1*)
FGSC11301 (*mat A*)
Round perithecia or very short beak

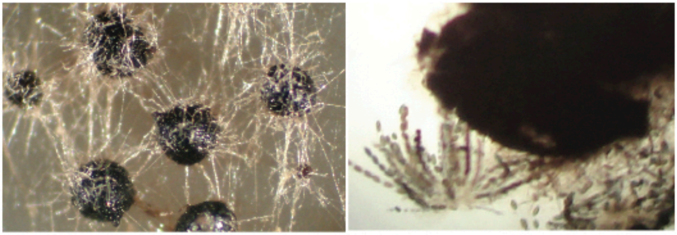

NCU09788 (*sbk-2*)
FGSC14570 (*mat a*)
FGSC14571 (*mat A*)
Small, short beak

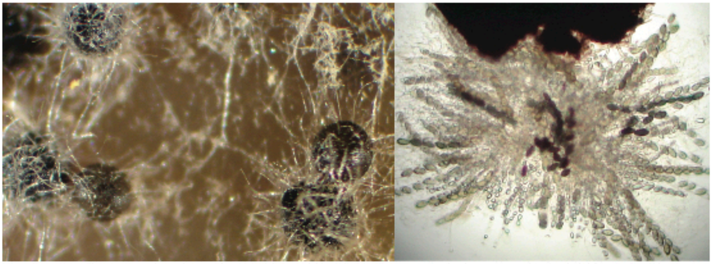

NCU09915
FGSC12534 (*mat A*)
Small, short beak

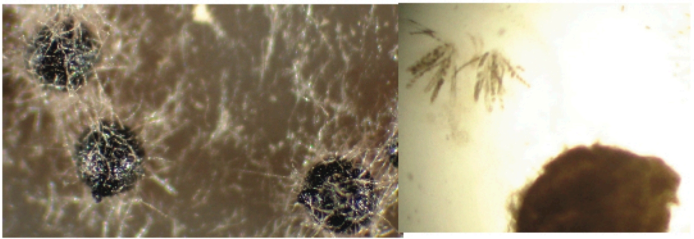

NCU07817 (*ncw-3*)
FGSC11687 (*mat a*)
FGSC11688 (*mat A*)
Protoperithecia only

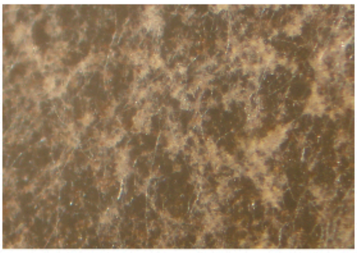

NCU07924
FGSC18242 (*mat A*)
Protoperithecia only

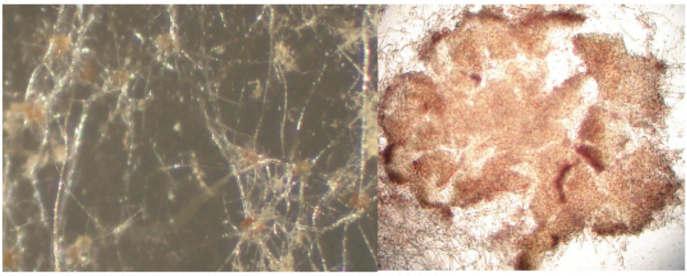

NCU08658
FGSC11059 (*mat a*)
FGSC21253 (*mat A*)
Protoperithecia only

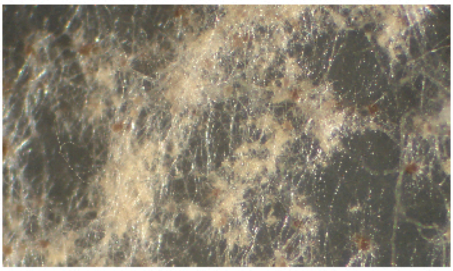

NCU09387 (*fmf-1*)
FGSC13451 (*mat a*)
FGSC13452 (*mat A*)
Protoperithecia only

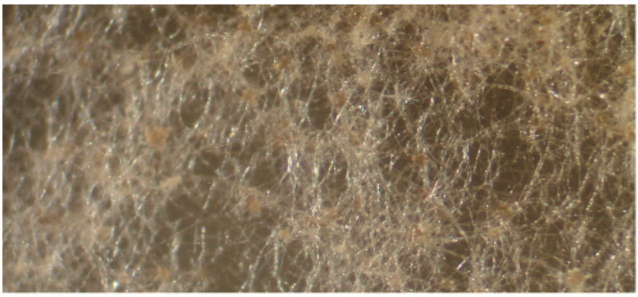

NCU09443
FGSC12514 (*mat a*)
Protoperithecia only

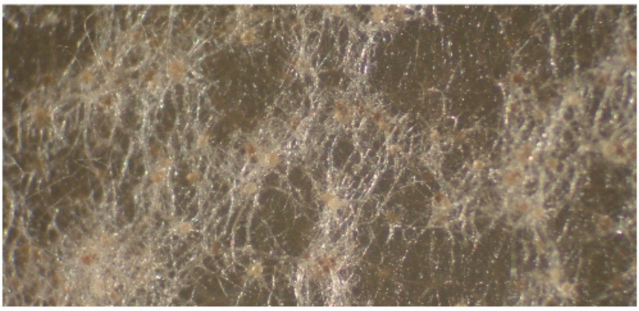

NCU02408
FGSC16221 (*mat a*)
Limited growth,
normal perithecia

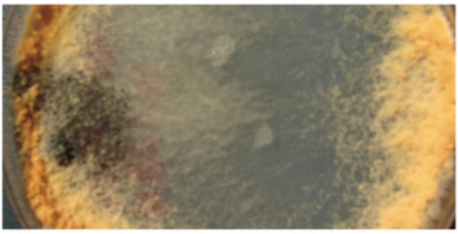

NCU04644
FGSC16528 (*mat a*)
Slow growth and
development,
normal perithecia

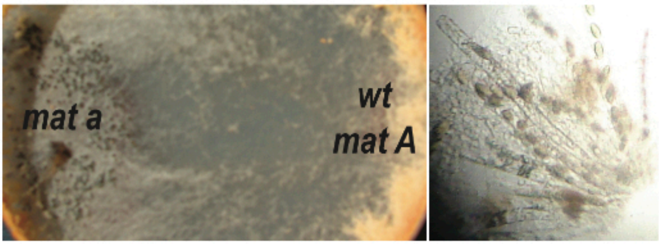

NCU08428

FGSC20312 (*mat a*)
FGSC20313 (*mat A*)
Larger perithecia;
slow developemnt
for *mat a* strain

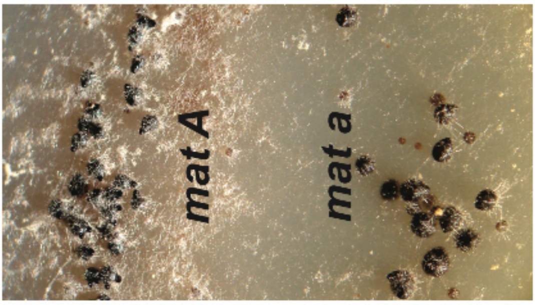

NCU01760

FGSC15499 (*mat A*)
Asci develop slowly,
few ascospores

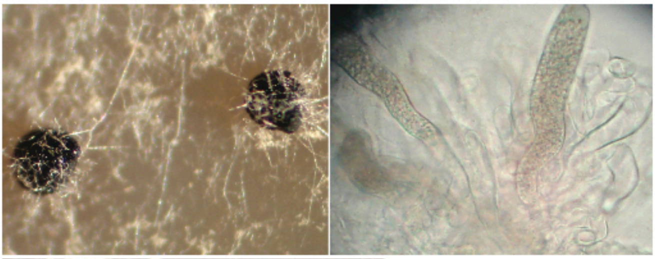

NCU03938
(*aod-5*)
FGSC11227 (*mat a*)
FGSC11228 (*mat A*)
Small, short beak,
some abortive
perithecia

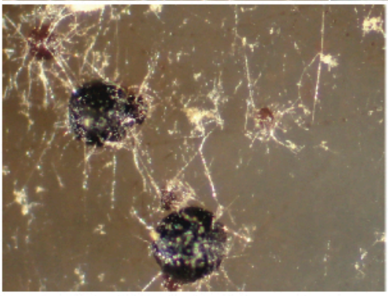

NCU01451
(*ipe-1*)
FGSC12844 (*mat a*)
FGSC12845 (*mat A*)
Abnormal beak;
no asci

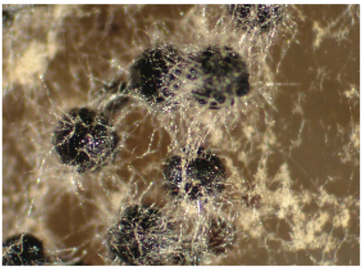

NCU01460

FGSC12798 (*mat a*)
FGSC12799 (*mat A*)
Round perithecia;
no asci

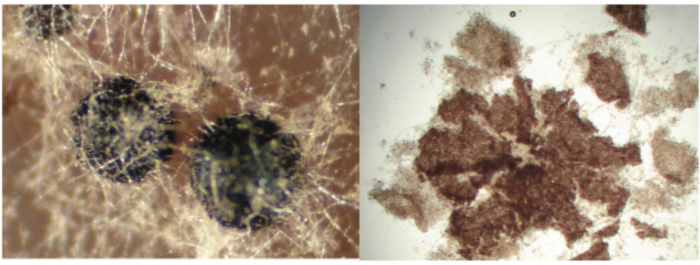

NCU01496
(*stc1*)
FGSC17464 (*mat a*)
FGSC17465 (*mat A*)
Short beak, no asci

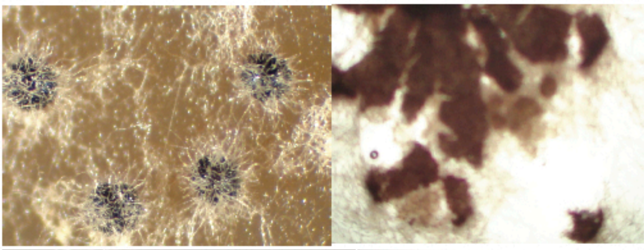

NCU06316
(*pna-2*)
FGSC20345 (*mat a*)
FGSC20346 (*mat A*)
No beak, no asci

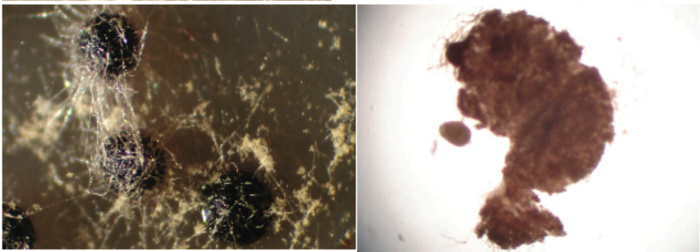

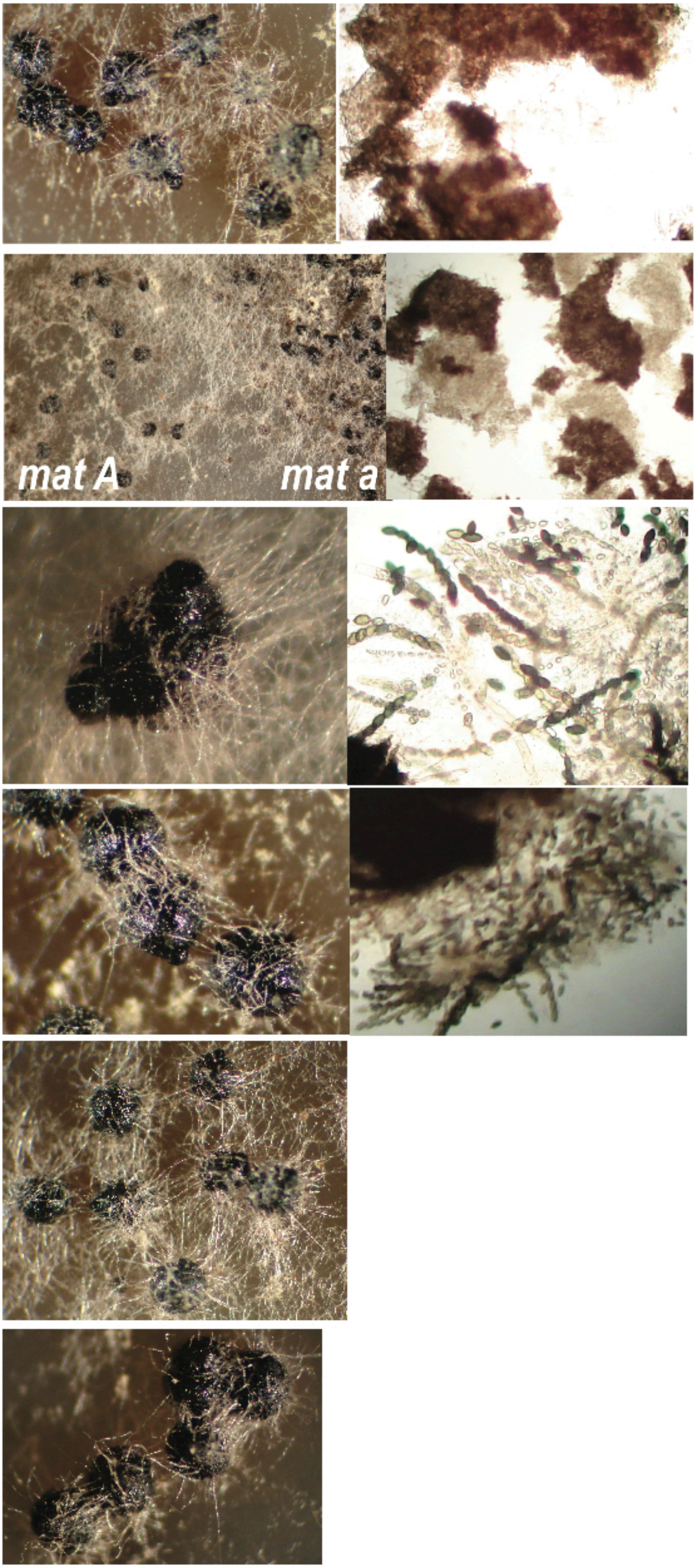

NCU07508

FGSC18105 (*mat a*)
FGSC18104 (*mat A*)
Beaked perithecia, no asci

NCU08856 (*pdv-1*)

FGSC18409 (*mat a*)
FGSC18410 (*mat A*)
Round perithecia; no asci

NCU00317

FGSC15867 (*mat A*)
Short, small beak

NCU01120 (*spo11*)

FGSC12440 (*mat a*)
FGSC12441 (*mat A*)
Short, small beak

NCU01134

FGSC12360 (*mat A*)
Short, small beak

NCU01640 (*rpn-4*)

FGSC15867 (*mat A*)
Short, small beak
