# Supplementary material for: The ancestral levels of transcription and the evolution of sexual phenotypes in filamentous fungi
Source: PLoS Genet. 2017 Jul 13;13(7):e1006867. doi: 10.1371/journal.pgen.1006867 (PMC5509106; doi:10.1371/journal.pgen.1006867)
Supplement: S3 Fig — (PDF) [file pgen.1006867.s003.pdf]

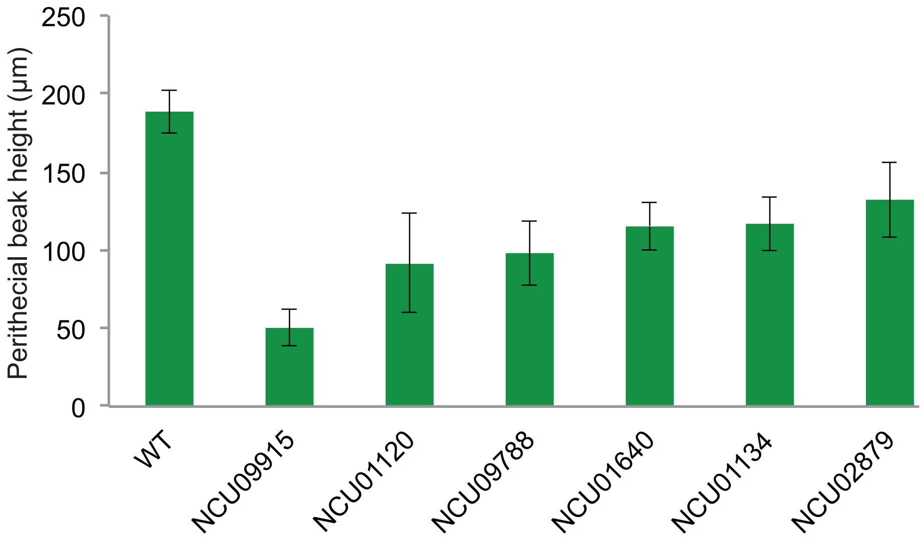

S3 Fig. Beak measurements for *N. crassa* wild type, and knockout mutants identified in this study as exhibiting short beaks. Measurement was conducted on 50 randomly selected perithecia. The error bar represents one standard deviation.
