# Supplementary material for: The ancestral levels of transcription and the evolution of sexual phenotypes in filamentous fungi
Source: PLoS Genet. 2017 Jul 13;13(7):e1006867. doi: 10.1371/journal.pgen.1006867 (PMC5509106; doi:10.1371/journal.pgen.1006867)
Supplement: S4 Fig — (PDF) [file pgen.1006867.s004.pdf]

	**(PH1 on left)**
PH-1	N/A
FGSG_0565	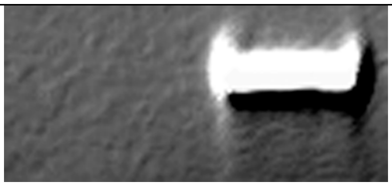<br>PH1: 0 bp<br>Δ: ≈ 1800 bp<br>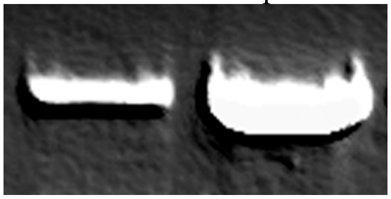<br>PH1: 3686 bp<br>Δ: 3656 bp
FGSG_1108	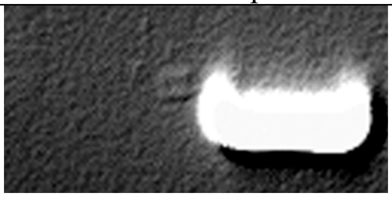<br>PH1: 0 bp<br>Δ: ≈ 1800 bp<br>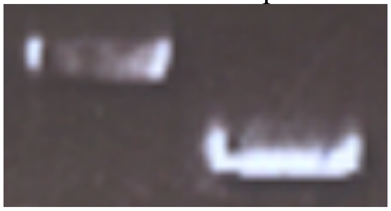<br>PH1: 4159 bp<br>Δ: 3600 bp

Figure S4. PCR verification of knockouts of *Fusarium graminearum.* Top image is of the internal check (*hyg* gene); Bottom image is the external check (primers from **Table S7**).

FGSG_2102	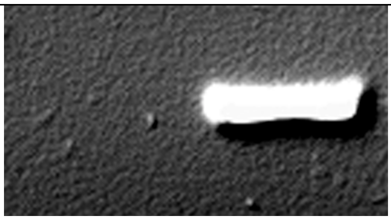<br>PH1: 0 bp<br>Δ: ≈ 1800 bp<br>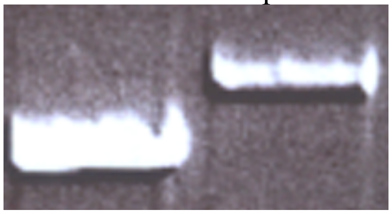<br>PH1: 2480 bp<br>Δ: 3630 bp
FGSG_3028	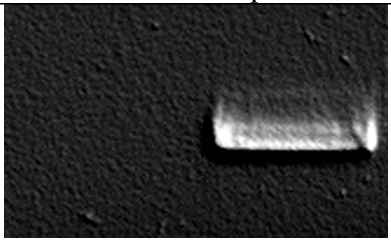<br>PH1: 0 bp<br>Δ: ≈ 1800 bp<br>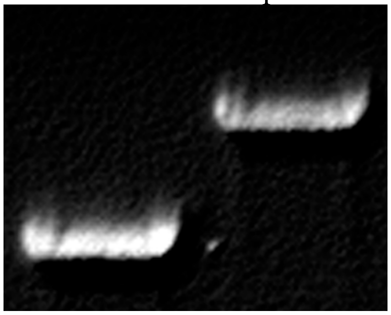<br>PH1: 2575 bp<br>Δ: 3882 bp
FGSG_4001	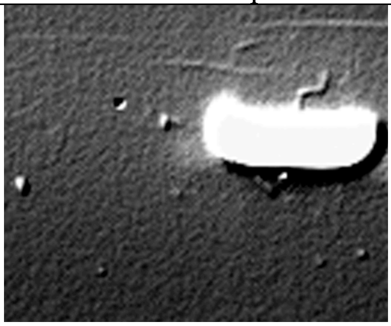<br>PH1: 0 bp<br>Δ: ≈ 1800 bp

	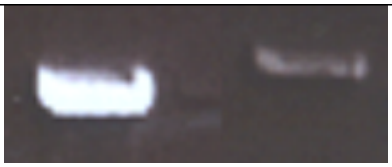<br>PH1: 2718 bp<br>Δ: 2752 bp
FGSG_4417	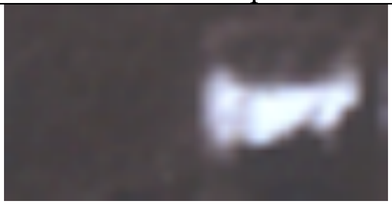<br>PH1: 0 bp<br>Δ: ≈ 1800 bp<br>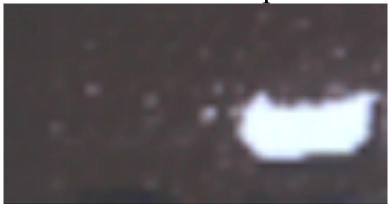<br>PH1: 0 bp<br>Δ: 2100 bp
FGSG_4997	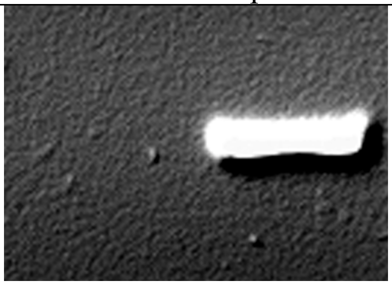<br>PH1: 0 bp<br>Δ: ≈ 1800 bp<br>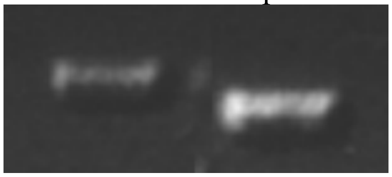<br>PH1: 3651 bp<br>Δ: 3599 bp
FGSG_5166	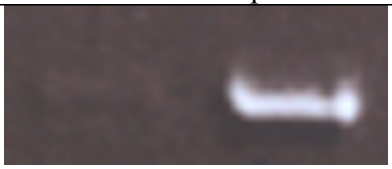<br>PH1: 0 bp<br>Δ: ≈ 1800 bp

	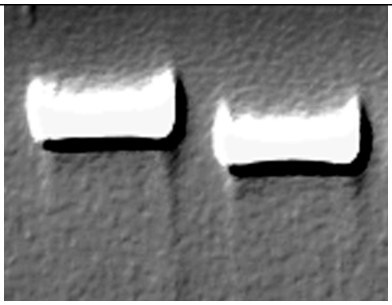<br>PH1: 3959 bp<br>Δ: 3525 bp
FGSG_5652	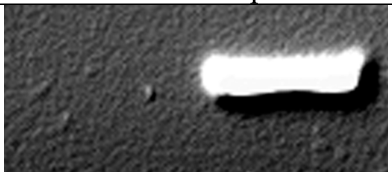<br>PH1: 0 bp<br>Δ: ≈ 1800 bp<br>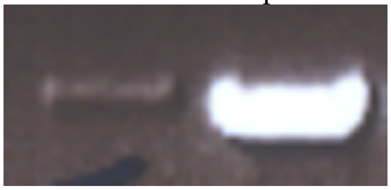<br>PH1: 4044 bp<br>Δ: 3606 bp
FGSG_6651	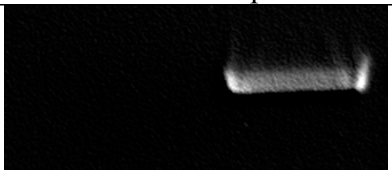<br>PH1: 0 bp<br>Δ: ≈ 1800 bp<br>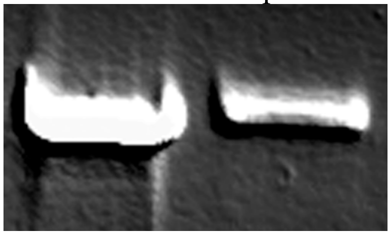<br>PH1: 3774 bp<br>Δ: 3930 bp
FGSG_7111	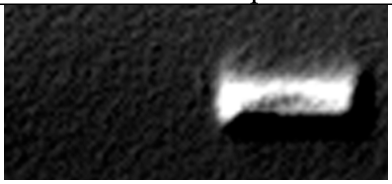<br>PH1: 0 bp<br>Δ: ≈ 1800 bp

	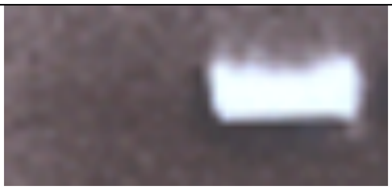<br>PH1: 0 bp<br>Δ: ≈ 1800 bp
FGSG_7478	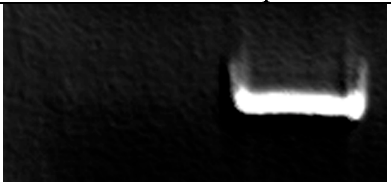<br>PH1: 0 bp<br>Δ: ≈ 1800 bp<br>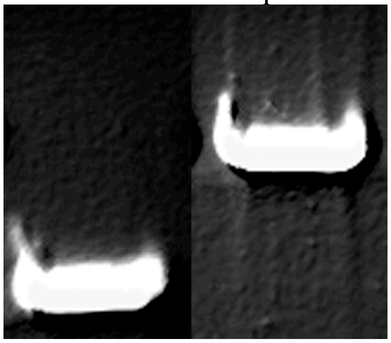<br>PH1: 2430 bp<br>Δ: 3771 bp
FGSG_8695	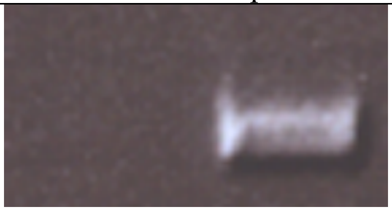<br>PH1: 0 bp<br>Δ: ≈ 1800 bp<br>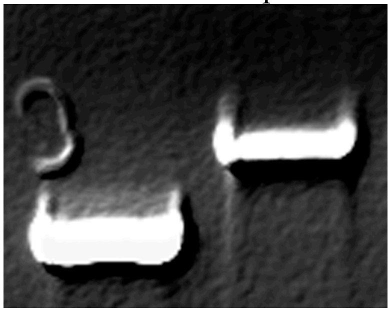<br>PH1: 2512 bp<br>Δ: 3552 bp

FGSG_10094	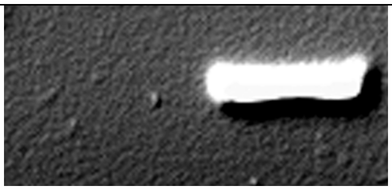<br>PH1: 0 bp<br>Δ: ≈ 1800 bp<br>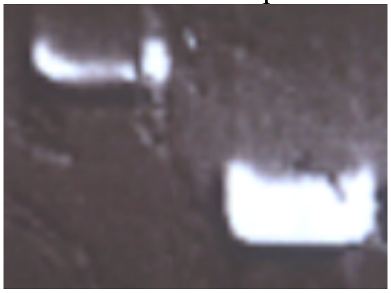<br>PH1: 6899 bp<br>Δ: 3612 bp
FGSG_13162	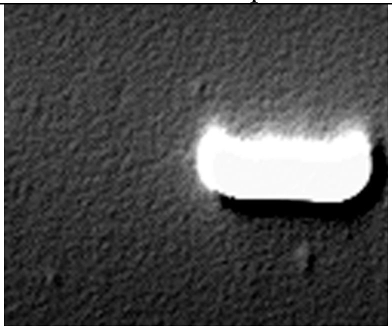<br>PH1: 0 bp<br>Δ: ≈ 1800 bp<br>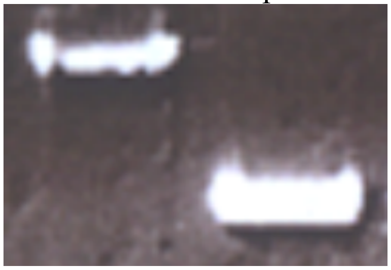<br>PH1: 7567 bp<br>Δ: 3414 bp
FGSG_16340	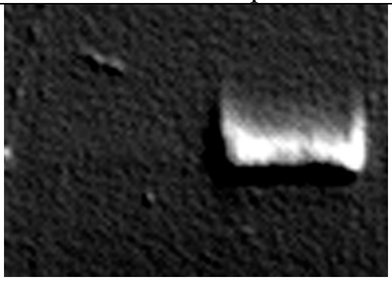<br>PH1: 0 bp<br>Δ: ≈ 1800 bp

	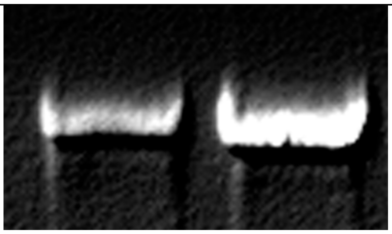<br>PH1: 3842 bp<br>Δ: 3777 bp
FGSG_16849	Photo Not Available<br>PH1: 0 bp<br>Δ: ≈ 1800 bp<br>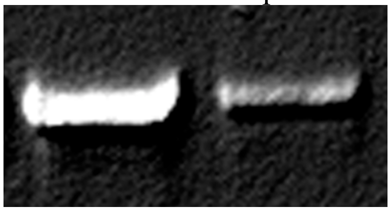<br>PH1: 3537 bp<br>Δ: 3738 bp
FGSG_17494	No Internal Check Completed<br>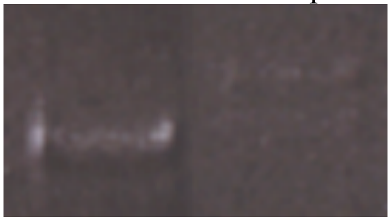<br>PH1: 7758 bp<br>Δ: 3941 bp
FGSG_17499	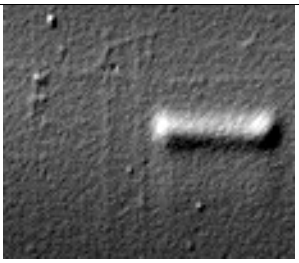<br>PH1: 0 bp<br>Δ: ≈ 1900 bp

	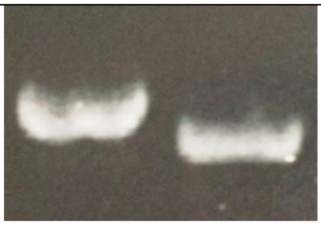<br>Δ: 3583 bp<br>PH1: 3160 bp
