# Supplementary material for: The ancestral levels of transcription and the evolution of sexual phenotypes in filamentous fungi
Source: PLoS Genet. 2017 Jul 13;13(7):e1006867. doi: 10.1371/journal.pgen.1006867 (PMC5509106; doi:10.1371/journal.pgen.1006867)
Supplement: S7 Table — (DOCX) [file pgen.1006867.s011.docx]

**Table S7**. Predicted networks of genes with phenotypes in perithecial development in *N. crassa* and *F. graminearum*.

| **Gene** | ***F. graminearum***  **(FGSG__)** | ***N. crassa***  **(NCU)** | ***F. graminearum***  **knockout phenotype** | ***N. crassa***  **knockout phenotype** |
| --- | --- | --- | --- | --- |
| ***Genes involved in early perithecial development*** | | | | |
| *div-12* | 00565 | 02496 | Few perithecia, delayed development | Few perithecia, no asci^a^ |
| *flbA* | 06228 | 08319 | Few perithecia, no maturation^a^ | Abnormal protoperithecia, no perithecia/ascospores^a^ |
| *fmf-1* | 01366 | 09387 | Perithecia/ no ascospores^a^ | Protoperithecia only^a^ |
| *lpe-1* | 07478 | 01451 | Few perithecia, delayed development | Abnormal ostioles, no ascospores |
| *lpe-2* | 05166 | 03490 | Few peritehcia, no asci | Wild type |
| *ncw-3* | 02190 | 07817 | n.d.^b^ | Protoperithecia only^a^ |
| *nor-1* | 04123 | 07850 | n.d. | Protoperithecia only^a^ |
| *pat-A* | 02386 | 08176 | n.d. | Early perithecia |
| *pdv-1* | 02751 | 08856 | Enhanced stroma | Arrest at early perithecial stage |
| *pls1* | 08695 | 07432 | Increased number of perithecia | Wild type |
| *pna-1* | 11826 | 08634 | Perithecia/ no ascospores^a^ | Perithecia/ no ascospores^a^ |
| *sdi-1* | 05164 | 03492 | n.d. | Protoperithecia only |
| *sdi-2* | 03069 | 04744 | n.d. | Protoperithecia only |
| *sdi-3* | 11184 | 05882 | n.d. | Protoperithecia only |
| *sdi-4* | 03028 | 04197 | Protoperithecia | Wild type |
| *vad-1* | 06651 | 00329 | Few perithecia, delayed development | Only vegetative and asexual development affected |
| ***Genes involved in late perithecial development*** | | | | |
| *aod-5* | 08626 | 03938 | Abnormal spores^c^ | Globose perithecia, short beak^c^ |
| *bek-1* | 05475 | 00097 | Few perithecia^c^ | No beak, no ascospores^a^ |
| *cch1* | 01364 | 02762 | No cirrhi, no firing^c^ | n.d. |
| *mid1* | 07418 | 06703 | No cirrhi, no firing^c^ | Wild-type spore release |
| *pna-2* | 04997 | 06316 | Stage 4 | No beak,  no asci/ascospores |
| *rel-1* | 04001 | 04520 | Stage 5 | Wild type |
| *rel-2* | 10094 | 01009 | Stage 5 | Wild type |
| *rel-3* | 01307 | 02724 | No firing^c^ | n.d. |
| *rel-4* | 17494 | 02908 | Stage 5 | Wild type |
| *rpn-4* | 04288 | 01640 | n.d. ^b^ | Short, small beak |
| *sbk-1* | 06331 | 02879 | n.d. | Short, small beak |
| *sbk-2* | 09475 | 09788 | n.d. | Short, small beak |
| *spo11* | 05949 | 01120 | Abnormal spores^c^ | Short small beak,  few dark ascospore^c^ |
| *stc1* | 04417 | 01496 | Stage 3 | Short beak,  no asci/ascospores^c^ |
| *tzn-1* | 02589 | 07621 | n.d. | Round perithecia,  small beak^c^ |

^a^ The genes *pna-1, fmf-1*, and *flbA* have been ascribed functions as transcription factors and knockout phenotypes have been previously described in *N. crassa* (Colot et al., 2006) and *F. graminearum* (Son et al., 2011). Phenotypes of *div-12, nor-1* and *ncw-3* in *N. crassa* were reported previously (Ghosh et al., 2014; Colot et al., 2006; Cano-Dominguez et al. 2008). Genes *pls1*, *vad-1* and *fmf-1* in *N. crassa* were also ascribed function in Borkovich et al. (2004).

^b^ n.d.: not determined.

^c^ Knockout phenotypes of *aod-5*, *bek-1*, *cch-1*, *mid1*, *rel-3*, and *spo11* in *F. graminearum* were reported in previous studies (Hallen et al., 2008; Cavinder et al., 2011; Son et al., 2011). Knockout phenotypes for genes *aod-5, bek-1, spo11, stc1*, and *tzn-1* in *N. crassa* were described in Borkovich et al. (2004) and Wang et al. (2014).
